# A mutation in F-actin polymerization factor suppresses the distal arthrogryposis type 5 PIEZO2 pathogenic variant in *Caenorhabditis elegans*

**DOI:** 10.1242/dev.202214

**Published:** 2024-02-13

**Authors:** Xiaofei Bai, Harold E. Smith, Luis O. Romero, Briar Bell, Valeria Vásquez, Andy Golden

**Affiliations:** ^1^ Department of Biology, University of Florida, Gainesville, FL 32610, USA; ^2^ Genetics Institute, University of Florida, Gainesville, FL 32610, USA; ^3^ National Institute of Diabetes and Digestive and Kidney Diseases, National Institutes of Health, Bethesda, MD 20892, USA; ^4^ Department of Physiology, College of Medicine, University of Tennessee Health Science Center, Memphis, TN 38103, USA; ^5^ Integrated Biomedical Sciences Graduate Program, College of Graduate Health Sciences, Memphis, TN 38163, USA

**Keywords:** Disease modeling in *C. elegans*, Distal arthrogryposis type 5, Forward genetic screening, Genetic suppressor, PIEZO channel, WAVE complex GEX-3

## Abstract

The mechanosensitive PIEZO channel family has been linked to over 26 disorders and diseases. Although progress has been made in understanding these channels at the structural and functional levels, the underlying mechanisms of PIEZO-associated diseases remain elusive. In this study, we engineered four PIEZO-based disease models using CRISPR/Cas9 gene editing. We performed an unbiased chemical mutagen-based genetic suppressor screen to identify putative suppressors of a conserved gain-of-function variant *pezo-1[R2405P]* that in human *PIEZO2* causes distal arthrogryposis type 5 (DA5; p. R2718P). Electrophysiological analyses indicate that *pezo-1(R2405P)* is a gain-of-function allele. Using genomic mapping and whole-genome sequencing approaches, we identified a candidate suppressor allele in the *C. elegans* gene *gex-3.* This gene is an ortholog of human *NCKAP1* (NCK-associated protein 1), a subunit of the Wiskott-Aldrich syndrome protein (WASP)-verprolin homologous protein (WAVE/SCAR) complex, which regulates F-actin polymerization. Depletion of *gex-3* by RNAi, or with the suppressor allele *gex-3(av259[L353F])*, significantly increased brood size and ovulation rate, as well as alleviating the crushed oocyte phenotype of the *pezo-1(R2405P)* mutant. Expression of GEX-3 in the soma is required to rescue the brood size defects in *pezo-1(R2405P)* animals*.* Actin organization and orientation were disrupted and distorted in the *pezo-1* mutants. Mutation of *gex-3(L353F)* partially alleviated these defects. The identification of *gex-3* as a suppressor of the pathogenic variant *pezo-1(R2405P)* suggests that the PIEZO coordinates with the cytoskeleton regulator to maintain the F-actin network and provides insight into the molecular mechanisms of DA5 and other PIEZO-associated diseases.

## Teaser

**Summary:** A forward genetic suppressor screen in *C. elegans* identifies a potential suppressor on *gex-3* , mitigating the reproductive defect induced by the DA5 mutation *piezo-1(R2405P)*.

## INTRODUCTION

PIEZO proteins are cation channels that transduce mechanical stimuli into physiological responses. PIEZO1 and PIEZO2 trimers form a propellor-like structure with C-terminal domains of each PIEZO monomer assembling to create the central ion pore (Saotome et al., 2018; Wang et al., 2019). Dysfunction in both human PIEZO1 and PIEZO2 causes a variety of physiological disorders and diseases in humans (Alper, 2017). Gain-of-function mutations in *PIEZO1* cause hereditary xerocytosis/dehydrated stomatocytosis (DHS) in red blood cells (Zarychanski et al., 2012; Albuisson et al., 2013; Glogowska et al., 2017; Andolfo et al., 2013), while loss-of-function mutations in *PIEZO1* could lead to congenital lymphatic dysplasia (Fotiou et al., 2015). In addition, some *PIEZO2* gain-of-function and loss-of-function mutations cause pathologies in distal limb and neuronal systems, which lead to joint contractures, muscle atrophy and proprioception deficits (Chesler et al., 2016; Coste et al., 2013; Ma et al., 2023). These pathogenic effects may occur due to insufficient or excessive responses of PIEZO channels to mechanical stimuli. At the cellular level, dysfunctional PIEZO channels may disrupt intracellular calcium homeostasis and interfere with downstream calcium-dependent signaling pathways (Alper, 2017; Beech and Kalli, 2019). Despite the electrophysiological characterization of PIEZO channel variants, at the functional level, the molecular and cellular mechanisms of PIEZO-associated diseases remain elusive.

The *C. elegans* spermatheca is a multicellular tube that can stretch and dilate. Oocytes enter the spermatheca through a distal neck, are fertilized within a central bag, and exit the spermatheca through the spermatheca-uterine (sp-ut) valve. The spermathecal valves and bag-cells are spatiotemporally coordinated to control oocyte entry during ovulation, as well as oocyte exit after fertilization in the spermatheca. The contractability of the spermathecal tissue is driven by the coordinated activation of myosin (Kelley and Cram, 2019; Kelley et al., 2020a,b). After ovulation, the fertilized oocytes are expelled from the spermatheca and into the uterus, pushing many sperm with them. These sperm must navigate back into the spermatheca for subsequent ovulations. The oocytes and somatic sheath cells secrete chemoattractant molecules and prostaglandins into the uterus to guide the sperm back to the spermatheca (Edmonds et al., 2010; Hoang et al., 2013). The *pezo-1* gene is the sole ortholog of human *PIEZO1* and *PIEZO2* in *C. elegans*. Dysfunctional PEZO-1 resulted in reduced brood size and low ovulation rate, as well as sperm guidance and navigational defects (Bai et al., 2020a). Cytoskeletal components play a crucial role in coordinating the contractibility of the spermathecal cells (Kelley and Cram, 2019; Kelley et al., 2020a,b). Furthermore, PIEZO channels activity is regulated by the cytoskeleton integrity (Atcha et al., 2021; Wang et al., 2022; Shmukler et al., 2015; Ellefsen et al., 2019; Eijkelkamp et al., 2013; Pardo-Pastor et al., 2018). Based on these findings, we hypothesize that the PIEZO channel plays a functional role during ovulation and contractility within the spermathecal tissue by regulating cytoskeletal activity, specifically actin polymerization. However, the exact role of PEZO-1 channel in governing the cytoskeletal activity during ovulation remains elusive.

In this study, we established a disease model of human PIEZO channels by generating orthologous variants in the *C. elegans pezo-1* gene using CRISPR/Cas9 gene editing. All homozygous *pezo-1* pathogenic variants reduced brood size and ovulation rate. Two of the mutants, *pezo-1(R2405L)* and *pezo-1(R2405P)*, caused sperm attraction defects, resulting in some of the sperm failing to migrate back to the spermatheca. Using a forward genetic screen, we found a suppressor allele in the gene *gex-3* that belongs to *NCKAP1* (NCK-associated protein 1), a subunit of Wiskott-Aldrich syndrome protein (WASP)-verprolin homologous protein (WAVE/SCAR) complex. Depletion of *gex-3* by RNAi and the suppressor allele *gex-3(L353F)* alleviated the small brood size and low ovulation rate, as well as the crushed oocyte phenotypes of the *pezo-1(R2405P)* mutant. Using an auxin-inducible degradation (AID) system, we drove tissue-specific degradation of GEX-3 in the *pezo-1(R2405P)* background. Reduced brood size was only restored in the somatic-specific degradation strain, suggesting that the loss of *gex-3* in somatic tissues primarily contributed to the suppression of the *pezo-1* gain-of-function mutation *(R2405P)*. Finally, we found that actin organization was disrupted and distorted in the *pezo-1* mutants, and that *gex-3(L353F)* partially alleviated actin defects in the *pezo-1(R2405P)* background. Overall, we used *C. elegans* to study the pathophysiological effect of an ortholog of human *PIEZO2* mutation causing distal arthrogryposis type 5 (DA5) and identified the putative suppressor *gex-3(L353F)*. Here, we provide new insights into the physiological roles of PIEZO channels and their interaction with cytoskeletal organizational elements.

## RESULTS

### PIEZO pathogenic variants cause reproductive deficiency in *C. elegans*

**Fig. 1.**
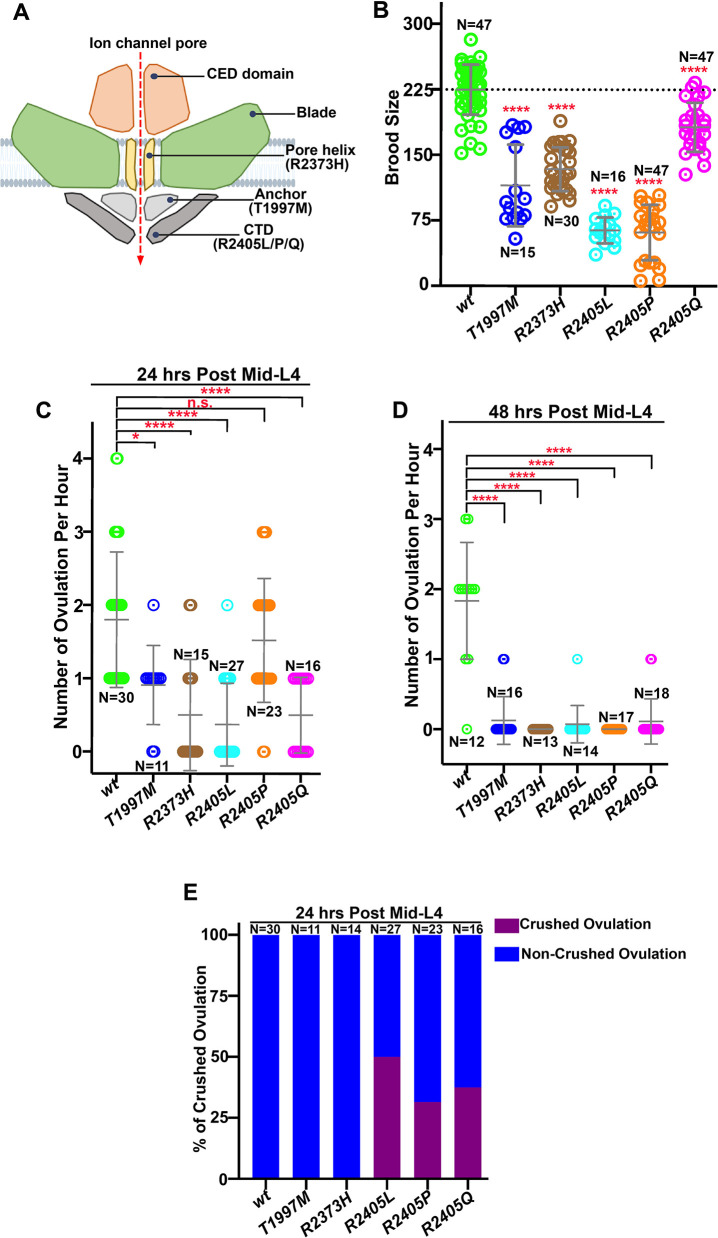
**PIEZO pathogenic variants caused reproductive deficiency in *C. elegans*.** (A) Diagram of the location of the PEZO-1 pathogenic residues in the PEZO-1 channel used in this study. (B) Brood size was reduced in all *pezo-1* pathogenic mutants tested when compared with wild type. *n* indicates the number of animals tested. (C-E) Quantification of the oocyte ovulation rate and percentage of crushed oocytes in wild type and *pezo-1* mutants during ovulation at different ages. *n* indicates the number of gonads tested. The oocyte ovulation rate was significantly reduced in the *pezo-1* mutant adults. One-way ANOVA test (B-D). *P=0.0114; ****P<0.0001 (unpaired one-way ANOVA *t*-test). Data are Data are median±s.d.

To study underlying molecular mechanisms of PIEZO-associated diseases, we used CRISPR/Cas9 gene editing to generate four disease-relevant PIEZO alleles in the C-terminal domains of *C. elegans pezo-1*, the sole worm ortholog of human *PIEZO1* and *PIEZO2* (Fig. 1A). Each of the alleles were orthologous mutations associated with three different diseases: dehydrated hereditary stomatocytosis (DHS), distal arthrogryposis subtype 5 disorder (DA5) and Gordon syndrome (GS) (Table 1). All homozygous *pezo-1* mutations caused a 27-81% reduction in brood size compared with the wild-type control (Fig. 1B). In particular, non-conservative amino acid residue mutations *pezo-1(R2405P)* and *pezo-1(R2405L)* decreased brood size by 70%, whereas a more conservative (polar) mutation, *pezo-1(R2405Q)*, decreased the brood size by ∼20% (Fig. 1B).

**Table 1.**
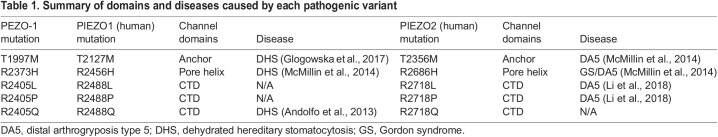
Summary of domains and diseases caused by each pathogenic variant

Our previous study demonstrated that the reduced brood size in *pezo-1* mutants was due to lower ovulation rate as well as oocyte crushing while exiting the spermathecal-uterine (sp-ut) valve (Bai et al., 2020a). To test whether ovulation was affected in each of the current *pezo-1* mutants, we performed live imaging to record the ovulation process for at least 1 h and analyzed the ovulation performance in each mutant. Indeed, we determined a low ovulation rate in all tested *pezo-1* mutants Fig. 1C,D). Of note, the ovulation rate was reduced to nearly zero in older animals (day-2 adults), whereas wild-type controls continued to ovulate ∼1.83 times per hour per gonad arm (Fig. 1D). We also observed that ∼30% of the ovulated oocytes from day 1 *pezo-1(R2405L/P/Q)* variants were crushed when transiting through the sp-ut valve, whereas no crushed oocytes were observed in the wild-type animals (Fig. 1E). Overall, our data suggest that *pezo-1* pathogenic variants caused a reduction in brood size, low ovulation rates and crushed oocytes.

### PIEZO pathogenic variants disrupt sperm guidance in *C. elegans*

**Fig. 2.**
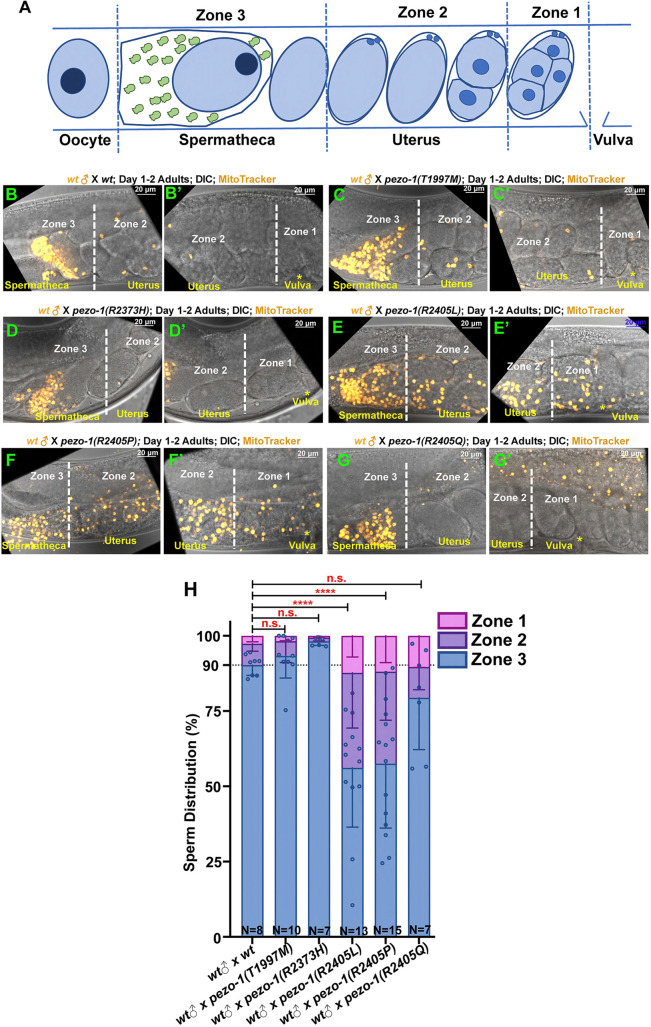
**Sperm guidance and navigation is disrupted in PIEZO pathogenic variants.** (A) To quantify sperm migration, sperm distribution was counted in three zones, including zone 3, which is the spermathecal region and the space containing the +1 fertilized embryo. Zone 1 is the area closest to the vulva; zone 2 is the area between zone 1 and zone 3. Sperm distribution is measured 1 h after the Mitotracker-labeled males were removed from the mating plate. (B-G′) The distribution of fluorescent male sperm (yellow dots) labeled with MitoTracker in the three zones in both wild type and *pezo-1* mutants. Yellow asterisks indicate the vulva (B′,C′,D′,E′,F′,G′). (H) Quantification of sperm distribution values for wild type and each *pezo-1* mutant. One-way ANOVA test. *n* indicates the number of animals tested. ****P<0.0001 (unpaired *t*-test). Data are median±s.d.

Various *pezo-1* null mutants exhibited defective sperm guidance (Bai et al., 2020a). Either self-sperm or male sperm failed to navigate back to the spermatheca after each ovulation, thus depleting the spermathecal sperm. To test whether each *pezo-1* missense mutant fails to attract sperm back to the spermatheca, male sperm navigational performance was assessed *in vivo* by staining wild-type male sperm with a vital fluorescent dye, MitoTracker CMXRos, which efficiently stains sperm mitochondria in live animals (Hoang et al., 2013). The stained wild-type males were placed for 30 min together with the label-free *pezo-1* mutant hermaphrodites. We isolated the hermaphrodites and allowed the sperm to navigate through the uterus to the spermatheca for 1 h. We quantified the sperm distribution by counting the number of fluorescent-labeled sperm in each zone (Fig. 2A-G) (Bai et al., 2020a). In wild-type hermaphrodites, 90.3% of fluorescent sperm navigated through the uterus and accumulated in the spermatheca (Fig. 2B,B′,H). The sperm distribution rate in the *pezo-1(T1997M)*, *pezo-1(R2373H)* and *pezo-1(R2405Q)* hermaphrodites were identical to wild-type worms [93.3% for *pezo-1(T1997M)*, 98.2% for *pezo-1(R2373H)* and 79.4% for *pezo-1(R2405Q)*] (Fig. 2C-D′,G-H). However, in the *pezo-1(R2405L)* and *pezo-1(R2405P)* reproductive tracts, the fluorescence-labeled sperm displayed defective sperm navigation. Only 56.1% and 57.5% of the labeled sperm in the *pezo-1(R2405L)* and *pezo-1(R2405P)* hermaphrodites, respectively, were able to migrate back to zone 3, which was the zone adjacent to and including the spermatheca (Fig. 2E-F′,H); the rest of the labeled sperm failed to reach the spermatheca and remained throughout zones 1 and 2, which are furthest from the spermatheca (Fig. 2E-F′,H).

### *pezo-1(R2405P)* is a gain-of-function allele

**Fig. 3.**
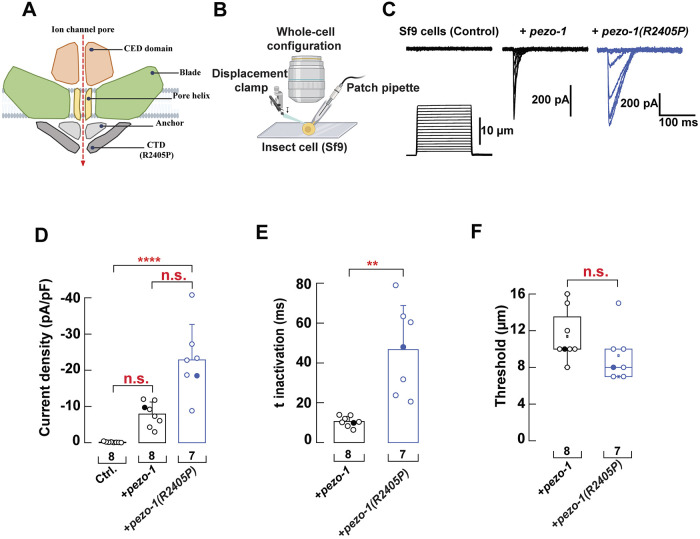
**Electrophysiological characterization of PEZO-1 gain-of function mutation *pezo-1(R2450P)*.** (A) Diagram of the location of the PEZO-1(R2405P) pathogenic residue in the PEZO-1 channel. (B) Schematic representation of the mechanical stimulation poked by a blunt pipette applied to Sf9 cells infected with baculovirus containing *pezo-1* (wild-type or R2405P constructs) recorded in the whole-cell configuration. Created with BioRender.com. (C) Representative whole-cell patch-clamp recordings (at −60 mV) of currents elicited by mechanical stimulation of Sf9 cells, uninfected (control), expressing *pezo-1* wild type or expressing *pezo-1(R2405P)*. Sf9 cells were poked with a heat-polished blunt glass pipette (3-4 µm) driven by a piezo servo controller. Displacement measurements were obtained with a square-pulse protocol consisting of 1 µm incremental indentation steps. Recordings with leak currents>200 pA and with access resistance>10 MΩ, as well as cells with giga seals that did not withstand at least five consecutive steps of mechanical stimulation were excluded from analyses. (D) Current density elicited by maximum displacement (−60 mV) of Sf9 cells expressing *pezo-1* wild type or *pezo-1(R2405P)*. Data are mean±s.d. Kruskal–Wallis (H=18.35; ****P=0.0001) and Dunn's multiple comparisons test. (E) Time constants of inactivation elicited by maximum displacement (−60 mV) of Sf9 cells expressing *pezo-1* wild type or *pezo-1(R2405P)*. Data are mean±s.d. Two-tailed unpaired *t*-test with Welch correction (t=−4.29). **P=0.0049. (F) Boxplots show the displacement thresholds required to elicit mechanocurrents of Sf9 cells expressing *pezo-1* wild type or *pezo-1(R2405P)*. Boxplots show mean (square), median (bisecting line), bounds of box (75th to 25th percentiles), outlier range with 1.5 coefficient (whiskers), and minimum and maximum data points. Two-tailed Mann–Whitney test (U=13.5). Filled circles come from the representative traces shown in C. *n* values are indicated under each column. Post-hoc *P*-values are indicated in the corresponding panels.

The *pezo-1(R2405P)* allele caused the most severe defects in the *C. elegans* reproductive tract (Figs 1 and 2). This mutation is equivalent to the mouse PIEZO1 R2514 mutation, which resides at the end of the α1 helix of the C-terminal domain. We determined that the longest isoform of *pezo-1* (isoform G; wormbase.org v. WS280) encodes a mechanosensitive ion channel (Millet et al., 2022). Of note, the behavior observed in the *pezo-1(R2405P)* mutant was similar to various *pezo-1* null mutants previously described (Bai et al., 2020a), suggesting that loss- or gain-of-function *pezo-1* mutations are equally pathogenic, similar to their human counterparts (Chesler et al., 2016; Coste et al., 2013; Ma et al., 2023). To determine the effect of *pezo-1(R2405P)* on PEZO-1 channel function, we heterologously expressed *C. elegans* wild-type and *pezo-1(R2405P)* mutant constructs in a *Spodoptera frugiperda* (Sf9) cell line and measured their mechanical response in the whole-cell patch-clamp configuration while stimulating with a piezo-electrically driven glass probe (Fig. 3A-C). Current densities for the *pezo-1(R2405P)* mutant were larger than non-infected cells, although not significantly different from wild-type *pezo-1* (Fig. 3D). Noteworthy, substituting an arginine with a proline at position 2405 results in mechanocurrents that become inactivated more slowly than the wild-type channel, as reflected by the large time constant of inactivation (Fig. 3E). We also determined that the R2405P construct requires less mechanical stimulation to open than wild-type *pezo-1* (Fig. 3F). Taken together, our results suggest that *pezo-1(R2405P)* is a gain-of-function allele.

### EMS-based forward genetic screen to identify suppressors of *pezo-1(R2405P)*

The small brood size in the *pezo-1* mutants was a simple readout to perform a forward genetic screen to isolate genetic suppressors of PIEZO disease mutations. To better understand the mechanism by which PEZO-1 regulates nematode reproduction, we conducted a genetic suppressor screen of the gain-of-function mutant *pezo-1(R2405P),* which has the lowest brood size of all the alleles we tested. Previously, we showed that *pezo-1* interacted genetically with an ER calcium regulator SERCA pump (*sca-1*) (Bai et al., 2020a). To increase the sensitivity of our forward screening, we shifted the synchronized EMS-treated F2 population of *pezo-1(R2405P)* embryos to *sca-1* RNAi food and maintained the animals at 25°C until the candidate suppressor lines were isolated (Fig. S1A). Under these sensitive screening conditions, wild-type hermaphrodites produced viable animals with a significant reduction in brood size (Fig. S1B), while *sca-1* RNAi treatment of our *pezo-1(R2405P)* mutant led to sterility or extremely low brood sizes (greater than 80% reduction compared with wild-type worms) after one or two generations (Fig. S1B). We screened ∼150,000 haploid genomes and isolated one stable suppressor line that partially restored the reduced brood size (*n*=161.1) when compared with *pezo-1(R2405P)* or *pezo-1(R2405P)* on *sca-1* RNAi food for one generation (*n*=87.4 and *n*=34.6, respectively) (total brood size=34.6) (Fig. S1B).

### MIP-MAP mapping and suppressor allele validation

Using a high-throughput genomic mapping strategy, which involves the molecular inversion probes genomic mapping (MIP-MAP) strategy (Mok et al., 2017), we identified genetic modifiers in the suppressor line *sup1* (Fig. S2). To maintain the *pezo-1(R2405P)* allele during the mapping process, we generated *pezo-1(R2405P)* in the mapping strain VC20019 background by CRISPR/Cas9 gene editing and named the strain *pezo-1(R2405P^MM^)* (Fig. S2A) (Mok et al., 2017). This strain appeared to have similar phenotypes to that of our original *pezo-1(R2405P)* mutant. We carefully pooled suppressed F2 progeny from the cross and allowed the F2 population to expand for ten generations for MIP-MAP analysis (Fig. S2A). As the flanking regions of the modifiers originated from a *pezo-1(R2405P)* background, the occurrence frequency of the MIP-MAP probes at the loci responsible for suppression would drop to nearly zero (Mok et al., 2017). Theoretically, reading the frequency of MIP-MAP probes would allow us to narrow down the target regions bearing the putative mutation. After MIP-MAP analysis and whole-genome sequencing, we mapped a single genomic region in the suppressor line *pezo-1(R2405P); sup1* (Fig. S2B), which contained candidate genes located on chromosome IV (9.1-12.0 Mb) (Fig. S2).

### A mutation in the WAVE regulatory complex NCKAP1 suppresses the reproductive defects caused by *pezo-1(R2405P)*

**Fig. 4.**
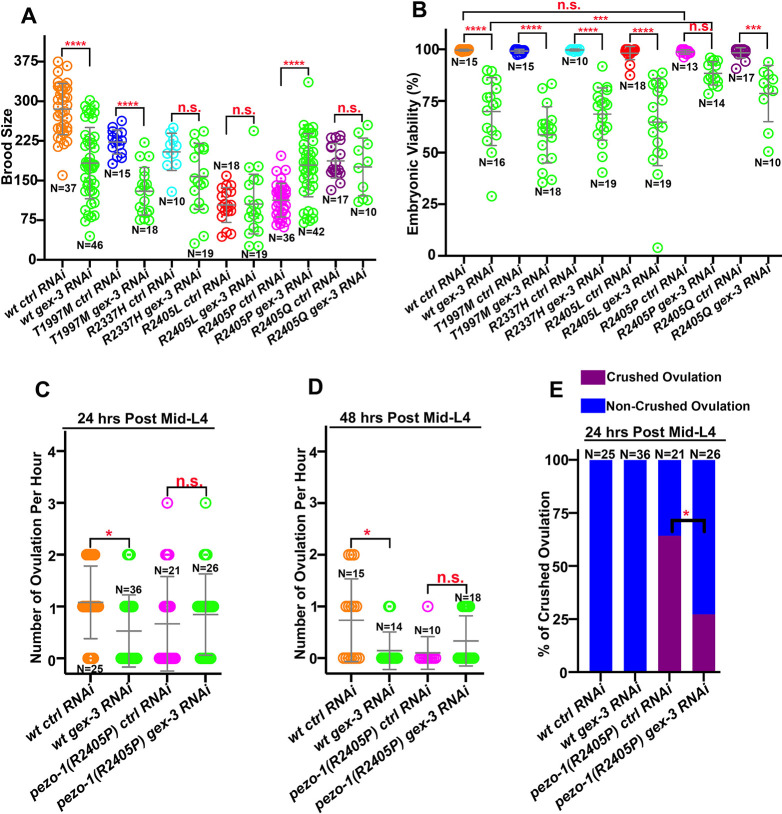
**The WAVE regulatory complex NCKAP1 suppresses the reproductive defects in the *pezo-1(R2405P)* mutant.** (A) *gex-3*(RNAi) treatment reduced brood size in wild-type and *pezo-1(T1997M)* mutant animals, while significantly restoring the brood size in *pezo-1(R2405P)* mutants. (B) Depletion of *gex-3* by RNAi led to various levels of embryonic lethality in all tested animals; however, the *pezo-1(R2405P)* allele partially alleviated the lethality when compared with wild-type control. *n* values indicate the number of animals tested in A and B. (C-E) Quantification of the oocyte ovulation rate and percentage of crushed oocytes of wild type and *pezo-1* mutants without or without *gex-3*(RNAi) treatment. *n* values indicate the number of gonads tested. One-way ANOVA (A-D) or Chi-squared-test (E). *P=0.0329 (C); *P=0.0247 (D), *P=0.0281 (E); ****P<0.0004 (*R2405P gex-3* versus *wt gex-3* RNAi in B); ***P=0.0007 (*R2405Q ctrl* versus *R2405Q gex-3* in B); ****P<0.0001 (A and B).

Assuming the candidate suppressors are loss-of-function alleles, we used RNAi to deplete expression of these candidate genes in the *pezo-1(R2405P)* mutant. From a RNAi trial experiment, we identified a candidate modifier gene, *gex-3*, on chromosome IV (Fig. 4A and Fig. S2B). *gex-3* is the ortholog of human *NCKAP1* (NCK-associated protein 1), one of the five WAVE regulatory complex subunits, which regulates the formation of the actin cytoskeleton via the Arp2/3 complex (Patel et al., 2008). To assess the knockdown efficiency of *gex-3* RNAi, we first fed *gex-3* (RNAi) bacteria to mNG::GEX-3 animals and quantified the fluorescence intensity of mNG::GEX-3 after 24 h of RNAi treatment (Fig. S3) (Dickinson et al., 2017). The mNG::GEX-3 levels in the germline and spermatheca were dramatically reduced when compared with RNAi-negative control (Fig. S3A-C), suggesting that *gex-3* RNAi effectively knocked down *gex-3* gene expression *in vivo*. We then depleted *gex-3* by RNAi in wild-type worms and all *pezo-1* pathogenic mutants (Fig. 4A,B). *gex-3*(RNAi) caused significant embryonic lethality in all tested strains (Fig. 4B); however, most embryos did hatch. *gex-3*(RNAi) also led to reduced brood sizes in both wild-type worms and the *pezo-1(T1997M)* mutant (Fig. 4A). The brood size in other tested mutants, including *pezo-1(R2373H)*, *pezo-1(R2405L)* and *pezo-1(R2373Q)*, were not significantly affected by *gex-3*(RNAi). Of note, the reduced brood size was significantly restored in *pezo-1(R2405P)* (*n*=113.1) by *gex-3*(RNAi) (*n*=179.3) (Fig. 4A). Additionally, depletion of *gex-3* by RNAi caused reduced embryonic lethality in the *pezo-1(R2405P)* mutant when compared with *gex-3* (RNAi) in the wild-type worms, indicating that *pezo-1(R2405P)* moderately suppressed the phenotype caused by *gex-3* (RNAi) (Fig. 4B). Furthermore, live imaging indicated that treatment with *gex-3* (RNAi) resulted in similar ovulation rates but fewer crushed oocytes in the *pezo-1(R2405P)* background (Fig. 4C-E). Using RNAi, we next tested whether other WAVE regulatory complex subunits suppressed the defects in the *pezo-1(R2405P)* mutant by depleting *abi-1* and *wve-1*, orthologs of human Abelson interactor gene (*ABI1*) and human Wiskott-Aldrich syndrome protein family member 1 gene (*WASF1*), respectively, using RNAi. However, the smaller brood size of the *pezo-1(R2405P)* mutant is not restored with either RNAi treatment (Fig. S4A,B).

### mNG::GEX-3 is expressed in multiple tissues and colocalizes with mScarlet::PEZO-1

**Fig. 5.**
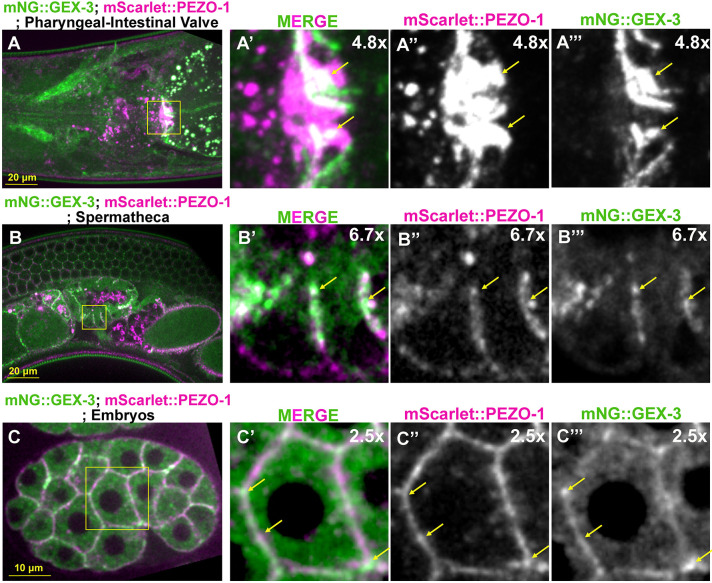
**mScarlet::PEZO-1 colocalizes with mNG::GEX-3 in multiple tissues and cells.** (A-A‴) mScarlet::PEZO-1 (magenta in A,A′) colocalizes with mNG::GEX-3 (green in A,A′) at the pharyngeal-intestinal valve. The enlarged pictures of the yellow rectangular area indicates the colocalization of mScarlet::PEZO-1 (magenta in A′) and mNG::GEX-3 (green in A′). (B-B‴) Colocalization of mScarlet::PEZO-1 (magenta in B,B′) and mNG::GEX-3 (green in B,B′) on the spermathecal membrane (yellow square in B). (B′-B‴) Higher magnification views of the area outlined in B showing colocalization of mScarlet::PEZO (magenta in B′) and mNG::GEX-3 (green in B′) on the membrane (yellow arrows). (C-C‴) mNG::GEX-3 and mScarlet::PEZO-1 was observed at the plasma membrane of the early embryos. Both mNG::GEX-3 (green in C′) and mScarlet::PEZO-1 (magenta in C′) were expressed on the embryonic plasma membrane (yellow arrows). Colocalization of mNG::GEX-3 and mScarlet::PEZO-1 appears white.

We used a CRISPR knock-in green fluorescent reporter mNeonGreen::GEX-3 (mNG::GEX-3) to determine the subcellular localization of GEX-3 in *C. elegans* (Dickinson et al., 2017). mNG::GEX-3 was widely expressed from embryonic stages to adulthood (Fig. 5). Of note, the mNG::GEX-3 was strongly expressed in various tissues subjected to mechanical stimulation, including the pharyngeal-intestinal valve and spermatheca (Fig. 5A-B‴). Notably, mNG::GEX-3 also co-localized with mScarlet::PEZO-1 at the pharyngeal-intestinal valve (Fig. 5A-A‴), spermathecal membrane (Fig. 5B-B‴) and early embryonic membranes (Fig. 5C-C‴).

### *gex-3(L353F)* suppresses the reproductive defects caused by *pezo-1(R2405P)*

**Fig. 6.**
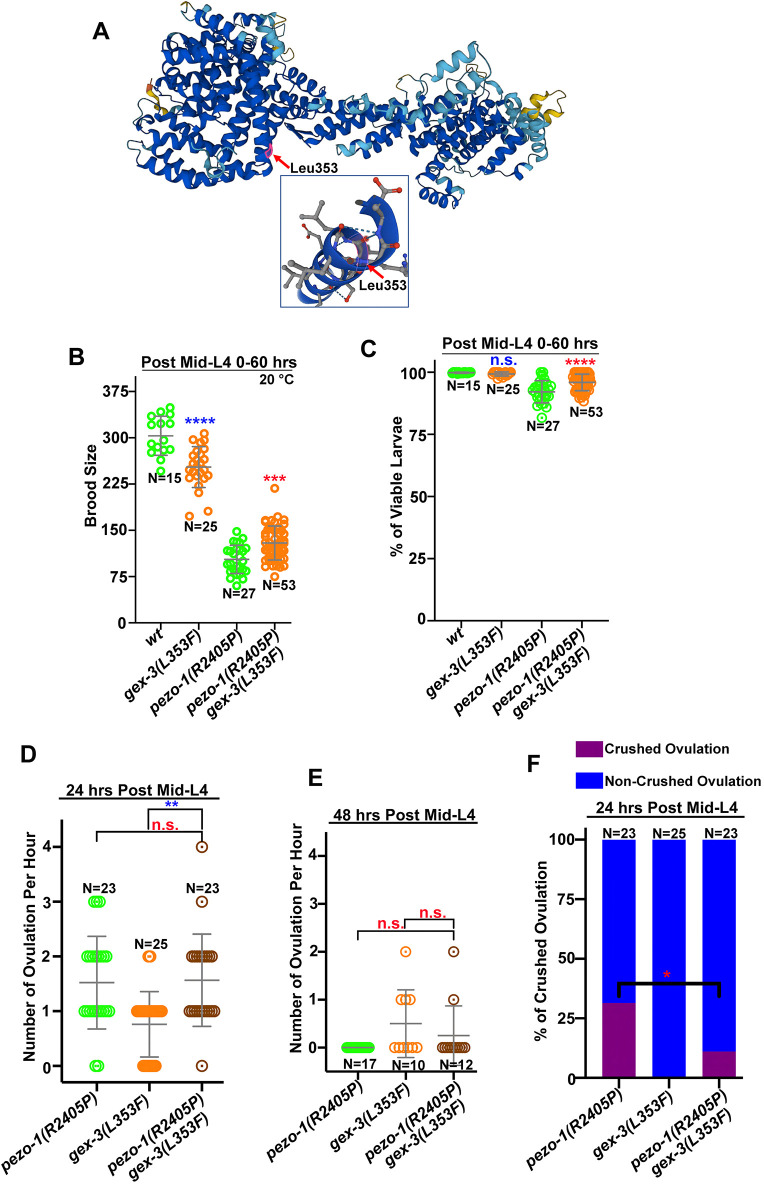
***gex-3(L353F)* suppresses the reproductive defects in the *pezo-1(R2405P)* mutant.** (A) The structure of GEX-3 from Alphafold indicated the Leu353 residue was on the N terminus of a helix (highlighted by red arrow). Different colors represent model confidence: dark blue, very high (pLDDT>90); cyan, confident (90>pLDDT>70); yellow, low (70>pLDDT>50); orange, very low (pLDDT<50). (B) *gex-3*(L353F) reduced brood size in wild-type animals but suppressed the smaller brood size in the *pezo-1(R2405P)* mutant animals. (C) The embryonic lethality in *pezo-1(R2405P)*, *gex-3(L353F)* and double mutants. *n* values in C indicate the number of animals tested in B and C. (D-F) Quantification of the oocyte ovulation rate and percentage of crushed oocytes of wild type, *pezo-1(R2405P)*, *gex-3(L353F)* and double mutants at different ages. *n* values indicate the number of the gonads tested. One-way ANOVA test (B-E) or Chi-squared-test (F). *P=0.0360 (F); **P=0.0015 (D); ***P=0.0008 (B); ****P<0.0001 (B and C).

After confirming that the of loss of function of *gex-3* suppressed the phenotypes in *pezo-1(R2405P)* animals, we generated a candidate suppressor allele by changing Leu353 to phenylalanine by CRISPR/Cas9 gene editing on the wild-type background. This allele was renamed as *gex-3(L353F)*. To assess its suppression during reproduction, this newly generated allele was then introduced onto the *pezo-1(R2405P)* background by CRISPR/Cas9 gene editing. The predicted GEX-3 structure from AlphaFold suggests that the residue Leu353 was located at the N terminus of a helical segment (Fig. 6A) (Varadi et al., 2022; Jumper et al., 2021). The homozygous *gex-3(L353F)* strain exhibited a smaller brood size when compared with wild-type worms (Fig. 6B). Unlike *gex-3(RNAi)* treatment, there was no embryonic lethality observed in the *gex-3(L353F)* mutant generated by CRISPR/Cas9 (Fig. 6C). To determine whether this missense mutation disrupted the temporal and/or spatial expression pattern of GEX-3, we generated the *gex-3(L353F)* allele using CRISPR/Cas9 on the mNG::GEX-3 reporter strain, which was named mNG::*gex-3(L353F)*. Endogenously tagged mNG::GEX-3 was expressed in multiple *C. elegans* tissues, including embryos, the spermatheca, the germline and pharynx (Fig. 5, Fig. S5A,C). Similar localization patterns were found in the mNG::*gex-3(L353F)* mutant (Fig. S5B,D). Overall, these data suggest that the *gex-3(L353F)* allele displays a weak effect, which may only partially compromise GEX-3 protein function, without altering the trafficking and cellular localization of GEX-3 *in vivo*.

The *gex-3(L353F); pezo-1(R2405P)* double mutant rescued the reduced brood size and subtle embryonic lethality observed in the *pezo-1(R2405P)* mutant alone (Fig. 6B,C). In addition, the double mutant reduced the crushed oocyte rate (from 31.4% to 11.1%) and slightly increased the ovulation rate to 0.25±0.62 ovulations per hour in the day 2 adults, when compared with *pezo-1(R2405P)* alone (0.0±0.0 UNITS) (Fig. 6D-F). The double mutant displayed a higher ovulation rate in day 1 animals (1.57±0.84 UNITS) than *gex-3(L353F)* alone (0.76±0.60 UNITS) (Fig. 6D), suggesting that *pezo-1(R2405P)* and *gex-3(L353F)* mutually suppress one another's reproductive defects.

The small brood size of the *pezo-1(R2405P)* mutant might arise from the high number of sperm that fail to navigate back to the spermatheca. To test whether the *gex-3(L353F)* allele restored sperm attraction back to the spermatheca, we assessed the MitoTracker-stained male sperm navigational performance in both *gex-3(L353F)* and *gex-3(L353F); pezo-1(R2405P)*. In *gex-3(L353F)* hermaphrodites mated with stained wild-type males, over 90% of fluorescent sperm navigated through the uterus and accumulated in the spermatheca (Fig. S6A,A′,C) within 1 h of mating. The sperm distribution rate in the double mutant hermaphrodites was 43.9%, which was similar to the *pezo-1(R2405P)* single mutant (57.5%, Fig. 2G), suggesting that *gex-3(L353F)* had no effect on sperm attraction behavior. Overall, our genetic interaction data suggest that both *gex-3* (RNAi) and the *gex-3(L353F)* mutant could partially suppress the reproductive defects of the *pezo-1(R2405P)* strain, but only at the ovulation and brood size levels. Meanwhile, the *pezo-1(R2405P)* allele also partially alleviated the embryonic lethality (Fig. 4B) and increased low ovulation rate (Fig. 4C) caused by *gex-3* (RNAi), suggesting that *pezo-1(R2405P)* and *gex-3* (RNAi) are mutual suppressors.

### Somatic tissue-specific degradation of GEX-3 suppresses the small brood size of the *pezo-1(R2405P)* mutant

GEX-3 is expressed in all reproductive tissues, including the spermatheca, germline, oocytes, and embryos (Fig. 5B,C). To better understand the role of GEX-3 in suppressing the subfertility of the *pezo-1(R2405P)* mutant, we used an auxin-inducible degradation system (AID) to degrade GEX-3 in somatic tissues or the germ line (Zhang et al., 2015). We knocked in a cassette with degron and GFP coding sequence at the *gex-3* N-terminus using CRISPR/Cas9 gene editing (named AID::GFP::GEX-3) so that the degron strain could be visualized and quantified by a GFP fluorescent signal (Fig. S7). The somatic- and germline-specific AID strains were driven by *Peft-3* and *Pmex-5,* respectively (Ashley et al., 2021), and activated when the animals were exposed to 2 mM auxin (indole-3-acetic acid, IAA). The GFP fluorescence intensities in the induced animals were significantly reduced compared to vehicle control (Fig. S7B-B″,D-D″,E,F). The strain expressing the degron interactor transgene *Pmex-5::tir-1::BFP::AID* led to a 2-3 fold reduction in fluorescence intensity of AID::GFP::GEX-3 in the germline and oocytes (Fig. S7B,B″,E), however, the intensity was not affected in the somatic tissues (Fig. S7E). The *Peft-3::tir-1::BFP::AID* led to an approximate 1.5 fold reduction of fluorescent intensity of AID::GFP::GEX-3 in the sheath, spermathecal cells, and germline (Fig. S7D,D″,F), suggesting the somatic degron strain affected the AID::GFP::GEX-3 in both somatic and germline cells.

**Fig. 7.**
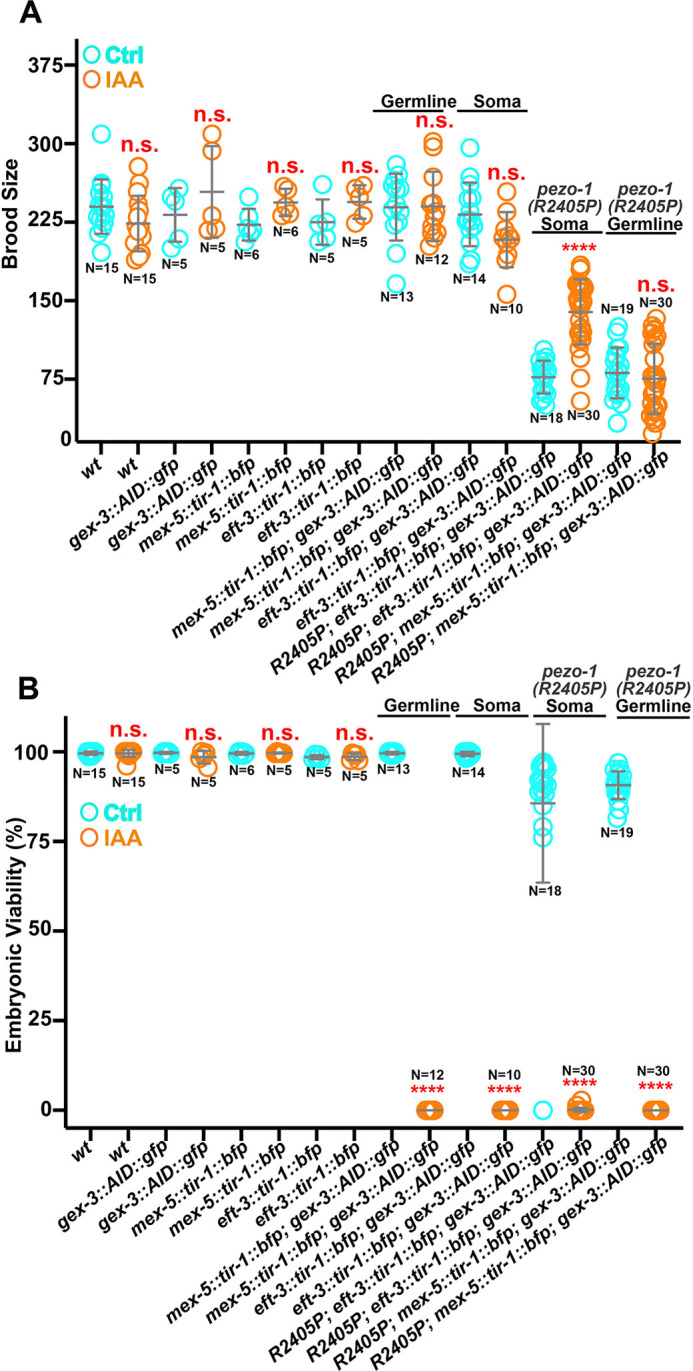
**Somatic tissue-specific degradation of GEX-3 suppresses the small brood size in the *pezo-1(R2405P)* mutant.** A degron and GFP cassette was inserted at the 5′ end of the *gex-3*-coding sequence using CRISPR/Cas9-mediated editing. The transgenic TIR-1::BFP::AID was driven by the *eft-3* promoter to be expressed in most or all somatic tissues, including the spermatheca and the somatic sheath cells. TIR-1::BFP::AID was driven by the germline-specific promoter *mex-5*, which drives expression in the germline and oocytes. (A) Brood size was partially restored in the *pezo-1(R2405P)* degron driven by the *eft-3* promoter when animals were treated with 2 mM auxin. (B) Embryonic viabilities were reduced to nearly zero in all degron strains when treated with 2 mM auxin. *n* values indicate the number of animals tested. ****P<0.0001 (unpaired, one-way ANOVA *t*-test). The blue circles represent those animals treated with the ethanol-only control; the orange circles represent those treated with auxin.

To assess the tissue-specific suppression of GEX-3 in *pezo-1(R2405P)*, we introduced the *pezo-1(R2405P)* allele into each degron strain by CRISPR/Cas9 gene editing. We exposed L4 animals to either 0.25% ethanol as a control or 1-2 mM IAA, then brood sizes and embryonic lethality rate were determined 0-60 h post-L4 (Fig. 7A,B). Interestingly, the brood size of the *pezo-1(R2405P)* mutant was significantly restored in the somatic tissue-specific AID::GFP::GEX-3 strain (Fig. 7A). Meanwhile, there were no significant changes in brood size in germline-specific AID::GFP::GEX-3 driven by the *Pmex-5* promoter (Fig. 7A). Additionally, depletion of GEX-3 in both somatic and germline tissues led to severe embryonic lethality (Fig. 7B). Unlike the partial rescue of embryonic lethality of *gex-3 (RNAi)* by *pezo-1(R2405P)*, embryonic lethality was close to 100% for *pezo-1(R2405P)*; AID::GFP::GEX-3 strains, regardless of the promoter used for the *tir-1::BFP::AID* cassette (Fig. 7B). Therefore, degradation of GEX-3 in the somatic tissue suppressed the small brood size of *pezo-1(R2405P)*, likely owing to the role of *gex-3* in the spermatheca or sheath contraction. This seems likely as we observed an improvement in ovulation performance when combining *gex-3* RNAi and *gex-3(L353F)* with *pezo-1(R2405P)* (Figs 4 and 6).

### Actin orientation and bundling are affected in *pezo-1* mutants

**Fig. 8.**
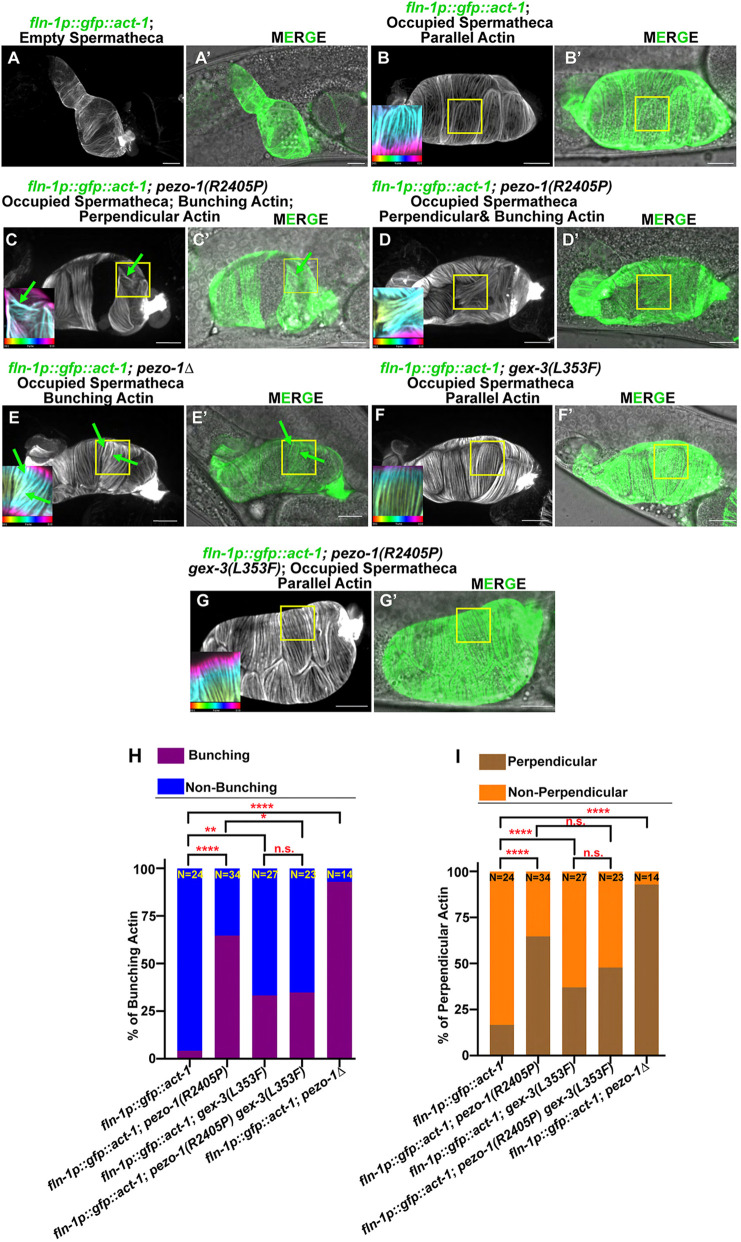
**Actin organization and orientation were disrupted in *pezo-1* mutants.** (A,A′) Representative images of the contracted spermatheca labelled by the actin marker GFP::ACT-1. (B,B′) Representative images of wild-type spermathecal cells with parallel actin bundles. (C-D′) Representative images of defective actin bundles, including perpendicular actin and bunching actin in the *pezo-1(R2405P)* mutant. Bunching actin is indicated by green arrows. (E,E′) Representative images of defective actin bundles in *pezo-1*Δ mutant. Bunching actin is indicated by green arrows. (F,F′) Actin organization in *gex-3(L353F)* animals. (G,G′) Double mutant *pezo-1(R2405P) gex-3(L353F)* suppressed the actin defects when compared with the *pezo-1(R2405P)* single mutant. The areas outlined in B-G′ are shown at higher magnification in the insets. (H,I) Quantification of actin defects in each strain. Insets in B,C,D,E,F,G are color coded according to *z*-depth to indicate the bundle organization and orientation. *n* values indicate the number of the occupied spermatheca tested in H and I. *P=0.0264 (H); **P=0.0088 (H); ****P<0.0001 (H and I) (Chi-squared test).

**Fig. 9.**
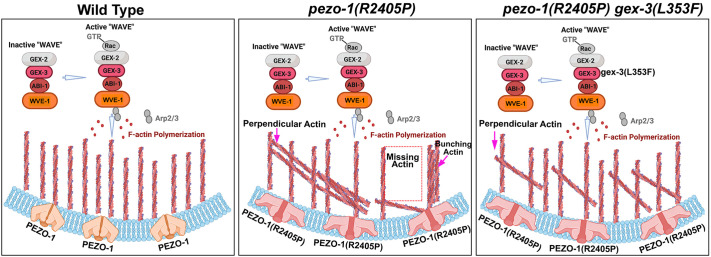
**Working model for genetic interaction between *pezo-1(R2405P)* and *gex-3(L353F)*.** The proposed model suggests how *gex-3(L353F)* or partial depletion of *gex-3* by RNAi might suppress the reproductive deficiency in the *pezo-1(R2405P)* mutant. At the cellular level, the *pezo-1(R2405P)* allele disrupts the actin organization and orientation, such as perpendicular actin and bunching actin in the spermathecal cells. The *gex-3(L353F)* allele partially alleviates the actin defects in the *pezo-1(R2405P)* mutant, which may explain its suppression of the low ovulation rate and number of crushed oocytes during ovulation, all leading to suppression of the reduced brood size caused by the *pezo-1(R2405P)* mutant. The proteins labeled in grey were not explored in this work.

The WAVE regulatory complex is essential for the organization of the actin cytoskeleton and its dynamics. Therefore, we predicted that *gex-3* may coordinate with *pezo-1* actin organization and orientation in the spermatheca. Previous studies indicated that spermathecal contractility was tightly associated with proper actin organization (Kelley and Cram, 2019; Kelley et al., 2020a,b). To further investigate the functional contribution of *pezo-1* and *gex-3* to spermathecal contractility and actin organization, we used a spermatheca-specific GFP marker GFP::ACT-1 for labeling actin. In mature wild-type animals, the entire actin network was tightly compacted in the contracting spermathecal cells (Fig. 8A,A′). In the dilated spermatheca occupied by an ovulating oocyte, each spermathecal cell contains prominent parallel actin bundles at the cellular cortex that run along the basal cell edges; these are known as peripheral actin bundles (Fig. 8B,B′). To test whether actin organization was affected by *pezo-1* and *gex-3*, we crossed the actin reporter strain into the following four strains: (1) *pezo-1(R2405P)*, (2) *pezo-1* full deletion mutant *pezo-1Δ*, (3) *gex-3(L353F)* and (4) the *pezo-1(R2405P) gex-3(L353F)* double mutant. We found a variety of defects in actin bundle distribution and orientation in the spermathecal cells of the *pezo-1(R2405P)* and *pezo-1Δ* mutants (Fig. 8C-E′,H,I). In these cells with actin bundle orientation defects, the actin bundles within the cells were aligned, but ran perpendicular to the long cell axis, termed perpendicular actin (Fig. 8C-D′,I). We also observed aligned actin that was densely associated with thicker and brighter actin bunches in *pezo-1(R2405P)* and *pezo-1Δ*, which were defined as bunched actin (Fig. 8D-E′,H) (Wirshing and Cram, 2018). The *gex-3(L353F)* mutant caused only mild actin defects, including bunching and perpendicular defects (Fig. 8F,F′,H). The *pezo-1(R2405P) gex-3(L353F)* double mutant significantly rescued bunching actin defects but did not affect perpendicular defects (Fig. 8G,G′,I). Collectively, these results indicate that *pezo-1* is crucial to spermathecal contractility, likely through influencing actin cytoskeletal organization and orientation. *gex-3(L353F)* partially suppressed the actin defects that may contribute to the alleviated ovulation defects and brood size (Fig. 9).

## DISCUSSION

The PIEZO proteins are associated with at least 26 human disorders and diseases (Alper, 2017). More than 100 variants of PIEZO have been identified to cause physiological disorders. Therefore, it is crucial to understand the molecular mechanism whereby dysfunctional PIEZO alters physiological processes, as well as to identify molecular and genetic determinants that may affect PIEZO activity. Complete knockout of *Piezo1* and *Piezo2* in mice causes embryonic lethality and fetal cardiac defects (Ranade et al., 2014; Nonomura et al., 2017). The PIEZO mutants in other systems, such as *Drosophila* or zebrafish, only lead to mild phenotypes (Kim et al., 2012; Duchemin et al., 2019; Shmukler et al., 2015), which limits the possibility of performing forward genetic screens to identify genetic determinants of PIEZO in those systems *in vivo*. Our previous study demonstrated that PEZO-1 channel influenced a series of reproductive processes, including ovulation, the expulsion of the fertilized oocyte into the uterus and sperm navigation (Bai et al., 2020a). Dysfunction of PEZO-1 causes a severe reduction in the ovulation rate, defective sperm navigation behavior and small brood size. The severe reduction in brood size in the *pezo-1* mutants provided an easy and reproducible readout for a forward genetic screen.

*Caenorhabditis elegans* is a powerful model in which gene editing and behavior are becoming an attractive system for precision modeling of human genetic diseases. In this study, we tested five PIEZO pathogenic variants in the *C. elegans pezo-1* gene, all of which displayed similar ovulation phenotypes as our *pezo-1* deletion mutants, suggesting that these alleles compromise PEZO-1 function and/or channel activity. All five variants were localized to the predicted pore of the PEZO-1 channel (Fig. 1A). The conserved human variants cause diseases in various organs or tissues, such as DHS in red blood cells or DA5, and Gordon syndrome in joint and distal extremities (Alper, 2017) (Table 1). The phenotypes observed in *C. elegans* do not reflect these PIEZO-derived disease symptoms, yet our disease modeling pipeline demonstrated the usefulness of the *C. elegans* reproductive tract for investigating the physiological contribution and molecular mechanisms of these PIEZO-based diseases mutations.

The severe reduction in brood size of the pathogenic mutants allowed us to screen putative genetic determinants for PIEZO suppressors *in vivo*. Our chemical mutagen-mediated forward genetic screening combined with MIP-MAP genomic mapping facilitated the discovery of the suppressor alleles. In this study, we successfully identified the cytoskeletal regulator WAVE/SCAR complex subunit GEX-3, which suppressed the defective phenotypes caused by the gain-of-function *pezo-1(R2405P)* allele. To our knowledge, this is the first genetic suppressor of PIEZO that has been identified through a forward genetic screening approach.

Actin-binding and regulatory proteins are crucial for proper spermathecal contractility and actin organization, which are necessary for achieving proper ovulation (Wirshing and Cram, 2018). Our actin imaging data revealed prominent parallel actin bundles, referred to peripheral actin bundles, at the basal cell surface (Fig. 9). In contrast, *pezo-1* mutants disrupted and distorted the actin organization and orientation in the spermatheca during ovulation (Fig. 9), likely contributing to the observed contractility and ovulation defects, such as crushed oocytes. PIEZO channel function relies on the effective communication and physical interactions between the channels and cytoskeletal components, including those between actin filaments and/or focal adhesions and the extracellular matrix (Wang et al., 2022; Ellefsen et al., 2019; Romero et al., 2020, 2023; Nourse and Pathak, 2017).

GEX-3 is a component of the WAVE complex, which controls actin cytoskeletal organization and dynamics by triggering the activity of the actin polymerization regulator Arp2/3 complex (Chen et al., 2010). Loss of *gex-3* resulted in a disrupted actin cytoskeleton during embryogenesis and axon migration in *C. elegans* (Bernadskaya et al., 2012). Based on our proposed model (Fig. 9), the GEX-3 suppressor allele could affect actin polymerization and actin organization in a way that partially alleviates the actin defects caused by the PEZO-1 gain-of-function mutation, thereby mitigating mechanotransduction deficiencies and improving spermathecal contractility during ovulation. These findings are consistent with recent studies showing that PIEZO activity could enhance actin polarization by physically tethering to the cadherin-β-catenin mechanotransduction complex (Atcha et al., 2021; Wang et al., 2022) (Fig. 9). Overall, the link between PEZO-1 and the actin cytoskeleton is likely part of a proposed feedback mechanism (Nourse and Pathak, 2017), in which the activation of PEZO-1 influences the dynamics and formation of cytoskeletal components, while the cytoskeleton affects PEZO-1 activation during mechanical activities such as ovulation.

In addition, the suppressor *gex-3* that we identified is likely to be *pezo-1(R2405)* allele specific, as it did not suppress the other four disease alleles generated in this study. We also could not rule out the possibility of an additional suppressor allele within the suppressor background, as we observed that the CRISPR-edited *gex-3* suppressor strain had weaker effects compared wit the original suppressor strain. Using this genetic screening pipeline will allow us to identify more suppressors in other pathogenic mutants, as well as uncover the molecular mechanism and functional contribution of each pathogenic allele to PEZO-1 activity. These screens may aid in defining the cellular mechanism that modulates PIEZO channel function and pave the way for further therapeutic approaches.

Finally, the human ortholog of *gex-3*, *NCKAP1*, plays a critical role in neurodevelopment. Disruptive variants of *NCKAP1* have been associated with neurodevelopmental disorders, including Coffin-Siris syndrome 1 (CSS1) and autism spectrum disorder (ASD) (Guo et al., 2020; Stelzer et al., 2016). The underlying causes of the *NCKAP1*-associated diseases are likely due to altered actin dynamics, which interfere with neuronal migration during cortical development (Guo et al., 2020). Patients with Coffin-Siris syndrome 1 variants exhibit symptoms such as aplasia or hypoplasia of the distal phalanges and abnormal facial features, which are similar to those observed in individuals with DA5 (Vergano and Deardorff, 2014). Therefore, by using genetic approaches, we can not only identify suppressors of disease relevant *pezo-1* mutants, but also uncover other molecules that contribute to these symptoms in humans. This helps to establish a molecular connection between disease-causing genes and provides valuable insights for future therapeutic advancements.

## MATERIALS AND METHODS

### *C. elegans* strains used in this study

*C. elegans* strains were maintained with Golden lab protocols (Bai et al., 2020b). Strain information is listed in Table S1.

### EMS suppressor screen

AG437 *pezo-1(R2405P)* early L4 hermaphrodites were washed three times in M9 and soaked in 48 mM ethyl methane sulfonate (EMS) solution for 4 h at room temperature. The EMS-treated animals were washed three times in M9 and were transferred to a fresh 100 mm MYOB plate with OP50 on one side. The animals were allowed to recover for up to 4 h before being picked to individual 100 mm MYOB plates with fresh OP50. Only the recovered animals that were able to crawl across the plates to the OP50 food were transferred to the fresh plates. A total of 70 MYOB plates with 25-30 mid-L4 (P0s) on each were incubated at 20°C. Gravid F1 adult were bleached and F2 embryos were collected after hypochlorite treatment. The F2 embryos were shaken in a glass flask with M9 buffer overnight, and hatched larvae were grown on *sca-1* RNAi plates at 25°C for 1 week. Their progeny were screened for viable larvae. Approximately 150,000 mutagenized haploid genomes were scored in this fashion.

### RNAi treatment

The RNAi feeding constructs were chosen from the Ahringer and Vidal libraries (Rual et al., 2004; Fraser et al., 2000). RNAi bacteria were grown until log phase was reached and spread on MYOB plates containing 1 mM IPTG and 25 μg/ml carbenicillin, and incubated for 12-14 h. The seeded RNAi plates were stored at 4°C up to 1 week. To deplete the target genes *gex-3*, *wve-1* and *abi-1*, mid-L4 hermaphrodites were picked onto plates with the IPTG-induced bacteria. Animals were grown on RNAi plates at 20°C for 36-60 h for brood size and other assays.

### Brood size determinations and embryonic viability assays

Single mid-L4 hermaphrodites were picked onto 35 mm MYOB plates seeded with 5-10 μl of fresh OP50 bacteria and allowed to lay eggs for 36 h (plate one contains the brood size from 0-36 h post mid-L4). The hermaphrodites were transferred to a newly seeded 35 mm MYOB plate to lay eggs for another 24 h and were flamed from the plate (the brood size on this plate was defined as the brood size from 36-60 h post mid-L4). Twenty-four hours after removing the hermaphrodites, the viable larvae were counted for the embryonic viability. Brood sizes were determined at 36 h and 60 h. Percentage of embryonic viability=(the number of hatched larva/the total brood size)×100.

### Live imaging to determine ovulation rates

For imaging ovulation, animals were immobilized on 7% agar pads with anesthetic (0.1% tricaine and 0.01% tetramisole in M9 buffer). DIC image acquisition was carried out using a Nikon 60× oil objective with 2-3 μm *z*-step size; 15-25 *z* planes were captured. Time interval for ovulation imaging is every 60-90 s, and duration of imaging is 60-90 min. Ovulation rate=(number of successfully ovulated oocytes)/total image duration. Actin imags were captured using a Nikon 60× water objective with 0.5 μm *z*-step size; 15-20 *z* planes were captured.

### CRISPR design

All CRISPR/Cas9 editing was generated into Bristol N2 strain as the wild type unless otherwise indicated. The crRNAs were synthesized from Horizon discovery, along with tracrRNA. Repair template and oligos were purchased from Integrated DNA Technologies (IDT). The CRISPR design followed the standard protocols (Paix et al., 2015). Approximately 20-30 young gravid animals were injected with the CRISPR/Cas9 injection mix. Detailed sequence information of CRISPR design is listed in Table S2.

### Sperm distribution assay and mating assay

MitoTracker Red CMXRos (MT) (Invitrogen, M7512) was used to label male sperm following the protocol adapted from previous studies (Hoang et al., 2013; Kubagawa et al., 2006). Wild-type males were incubated in the MT buffer for 2 h in the dark. The stained males were covered with foil to prevent light exposure overnight. About 30 males were placed with 10 anesthetized hermaphrodites (0.1% tricaine and 0.01% tetramisole in M9 buffer) on MYOB plates seeded with a 10 μl OP50 bacteria. After 20-30 min of mating, hermaphrodites were then isolated and allowed to rest on food for at least 1 h. The mated hermaphrodites were then mounted for microscopy on 7% agarose pads with the anesthetic. Image acquisition was carried out using a Nikon 60×1.2 NA water objective with 1 μm *z*-step size. Sperm distributions were quantified using the ImageJ cell counter.

### Auxin-inducible treatment in the degron strains

Auxin indole-3-acetic acid (IAA) was purchased from Alfa Aesar (A10556). A 400 mM stock solution of IAA was made in ethanol and was added to MYOB medium to a final concentration of 1 or 2 mM auxin. To efficiently degrade the GEX-3 protein, mid-L4 hermaphrodites were picked onto auxin plates. Animals were grown on the plates at 20°C for 24 h for the degradation efficiency test, and for 60 h for brood size assay.

### Microscopy

All imaging was carried out on a spinning disk confocal system that uses a Nikon 60×1.2 NA water or oil objectives, a Photometrics Prime 95B EMCCD camera and a Yokogawa CSU-X1 confocal scanner unit. Nikon's NIS imaging software were applied to capture the images. The image data were processed using ImageJ/FIJI Bio-formats plug-in (National Institutes of Health) (Schindelin et al., 2012; Linkert et al., 2010).

### Expression of *pezo-1* in Sf9 insect cells

To express PEZO-1 in Sf9 cells (a clonal isolate of *Spodoptera frugiperda* Sf21 cells), we generated recombinant baculoviruses, according to the manufacturer's instructions (Bac-to-Bac expression system; Invitrogen). To generate this baculovirus, we used a pFastBac construct (Epoch Life Science) containing an 8× histidine–maltose binding protein tag and a synthesized *pezo-1* isoform G nucleotide sequence (one of the longest isoforms according to RNA sequencing; wormbase.org release WS280). For expression of PEZO-1 R2405P, the construct contained an 8× histidine–maltose binding protein tag and a synthesized *pezo-1* isoform G with the R2405P point mutation. We infected Sf9 cells with either wild-type or mutant *pezo*-*1* baculovirus for 48 h as previously described (Millet et al., 2022). Infected cells were plated on glass coverslips coated with a peanut lectin solution (1 mg/ml; Sigma-Aldrich) for patch-clamp experiments.

### Electrophysiology and mechanical stimulation

Control and *pezo-1* (wild-type or R2405P constructs) infected Sf9 insect cells were recorded in the whole-cell patch-clamp configuration, as previously described (Millet et al., 2022). The bath solution contained 140 mM NaCl, 6 mM KCl, 2 mM CaCl_2_, 1 mM MgCl_2_, 10 mM glucose and 10 mM HEPES (pH 7.4). The pipette solution contained 140 mM CsCl, 5 mM EGTA, 1 mM CaCl_2_, 1 mM MgCl_2_ and 10 mM HEPES (pH 7.2). Sf9 cells were mechanically stimulated with a heat-polished blunt glass pipette (3-4 µm) driven by a piezo servo controller (E625; Physik Instrumente). The blunt pipette was mounted on a micromanipulator at an ∼45° angle and positioned 3-4 µm above the cells without indenting them. Displacement measurements were obtained with a square-pulse protocol consisting of 1 µm incremental indentation steps, each lasting 200 ms, with a 2 ms ramp in 10 s intervals. Recordings with leak currents over more than 200 pA and with an access resistance of more than 10 MΩ, as well as cells with giga-seals that did not withstand at least five consecutive steps of mechanical stimulation, were excluded from analyses. Pipettes were made from borosilicate glass (Sutter Instruments) and were fire-polished before use until a resistance between 3 and 4 MΩ was reached. Currents were recorded at a constant voltage (−60 mV), sampled at 20 kHz, and low-pass filtered at 2 kHz using a MultiClamp 700B amplifier and Clampex (Molecular Devices). Leak currents before mechanical stimulation were subtracted offline from the current traces. The time constant of inactivation τ was obtained by fitting a single exponential function, Eqn 1, between the peak value of the current and the end of the stimulus:
(rmEqn1)

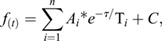
where A=amplitude, τ=time constant and the constant y-offset=*C* for each component i.

### MIP-MAP and data analysis

*pezo-1(R2405P^MM^)* males were mated with the homozygous hermaphrodites of each suppressor line (Fig. S2A). We pooled suppressed F2 progeny from the cross and allowed the F2 population to expand for ten generations for MIP-MAP analysis. Ten generations of self-recombination are sufficient to distribute the MIP-MAP single nucleotide polymorphism (SNPs) (also refer to molecular probes) into the suppressor line background and provide a high molecular resolution to identify the mutated regions. Candidate mutations (defined as novel, homozygous and nonsynonymous) were identified by whole-genome sequencing as described previously (Smith, 2022). Briefly, sequencing libraries were constructed using a Invitrogen Pure Link Genomic DNA Mini Kit (K1820-01) with genomic DNA from homozygous suppressor-bearing strains. The libraries were pooled and sequenced on a HiSeq 3000 instrument (Illumina) to at least 20-fold coverage. Variants were identified with a pipeline of BBMap (Bushnell, 2022), SAMtools (Li et al., 2009), FreeBayes (Garrison and Marth, 2012) and ANNOVAR (Wang et al., 2010). Mapping loci for suppressors were identified using molecular inversion probes (MIPs) to SNPs as described previously (Mok et al., 2017). Briefly, suppressor-bearing strains were mated to SNP mapping strain VC20019 (Thompson et al., 2013), which had been engineered via CRISPR to contain the *pezo-1(R2405P)* mutation. F1 cross-progeny were allowed to self-fertilize, and a minimum of 50 homozygous F2 progeny were pooled for construction of MIP libraries. SNP allele frequencies were determined using a custom script and plotted with R (R Core Team, 2021) to delimit the mapping interval.

### Data and statistical analyses

The electrophysiological data and statistical analyses were performed using GraphPad Instat 3 and OriginPro 2018 software. Statistical methods and sample numbers are detailed in the corresponding figure legends. No technical replicates were included in the analyses. Statistical significance for other assays was determined by the *P*-value from an unpaired two-tailed *t*-test, one-way ANOVA or Chi-squared test. ns=not significant; *P<0.05; **P<0.01, ***P<0.001; ****P<0.0001. Both the Shapiro-Wilk and Kolmogorov–Smirnov Normality test indicated that all data follow normal distributions.

## Supplementary Material



10.1242/develop.202214_sup1 Supplementary information
