# Supplementary material for: A mutation in F-actin polymerization factor suppresses the distal arthrogryposis type 5 PIEZO2 pathogenic variant in Caenorhabditis elegans
Source: Development. 2024 Feb 13;151(4):dev202214. doi: 10.1242/dev.202214 (PMC10911111; doi:10.1242/dev.202214)
Supplement: Supplementary information [file develop-151-202214-s1.pdf]

Supplemental Figure 1

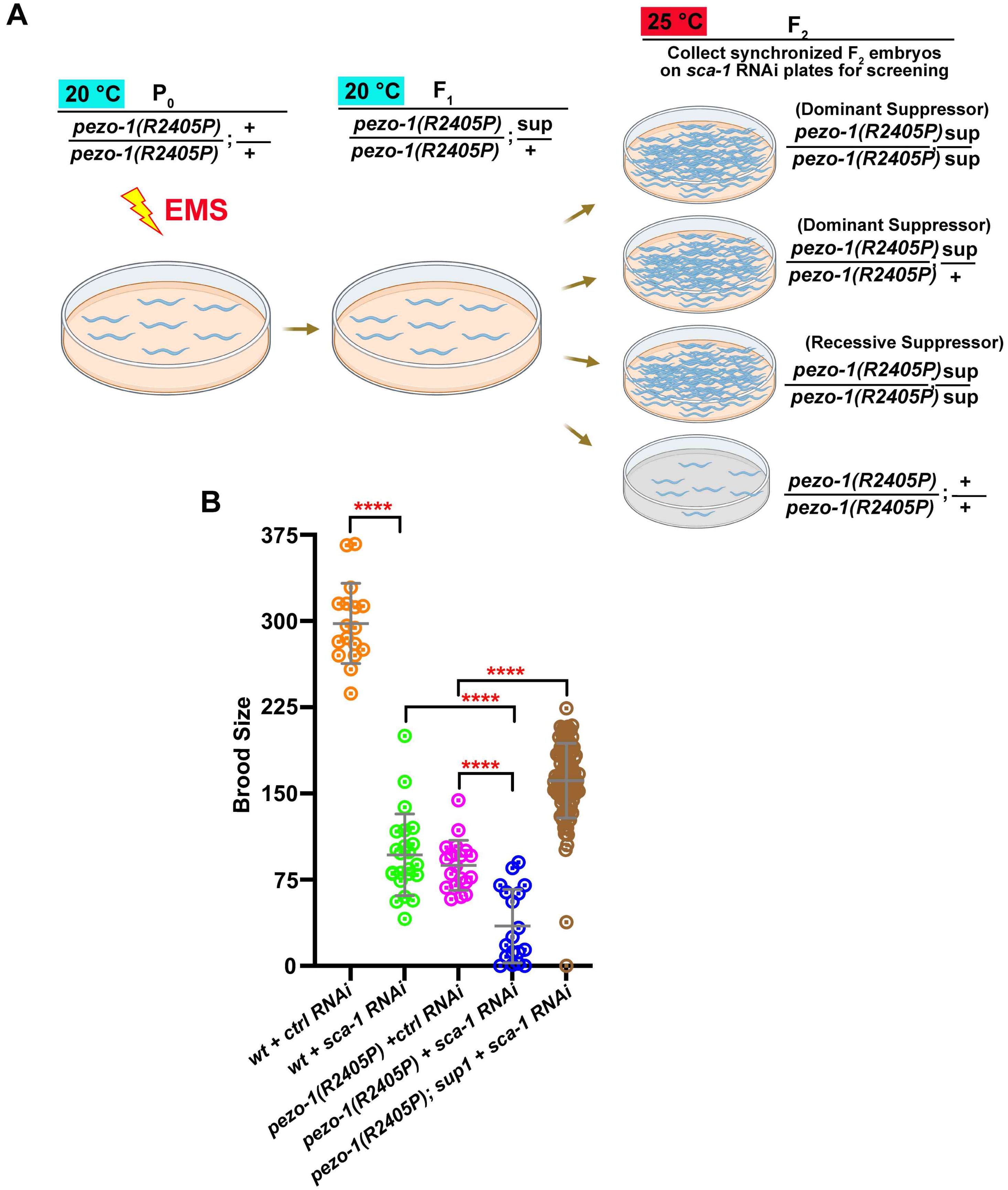

Development • Supplementary information

Fig. S1. Forward genetic screen for suppressors of the small brood size of a DA5 patient-specific allele *pezo-1(R2405P)*

(A) Strategy to identify genetic modifiers that restore the small brood size of *pezo-1(R2405P)* mutants. The synchronized L4 larvae of the *pezo-1(R2405P)* mutation (P0) were collected and treated with EMS. The healthy EMS-treated late L4 larvae were picked to a single plate to propagate F1 populations at 20°C. The gravid F1 adults were bleached, and the viable F2 larvae were transferred to *sca-1* RNAi plates at 25°C. These F2 animals were allowed to grow and were screened for fertility and restored brood sizes. (B) Brood of wild type, *pezo-1(R2405P)*, and the suppressor line at different conditions over 60 hours post mid-L4. N values indicated the number of the tested animals in (B).. P-values: ****, p<0.0001 (t-test). The graphic was generated with BioRender.com.

Development • Supplementary information

Supplemental Figure 2

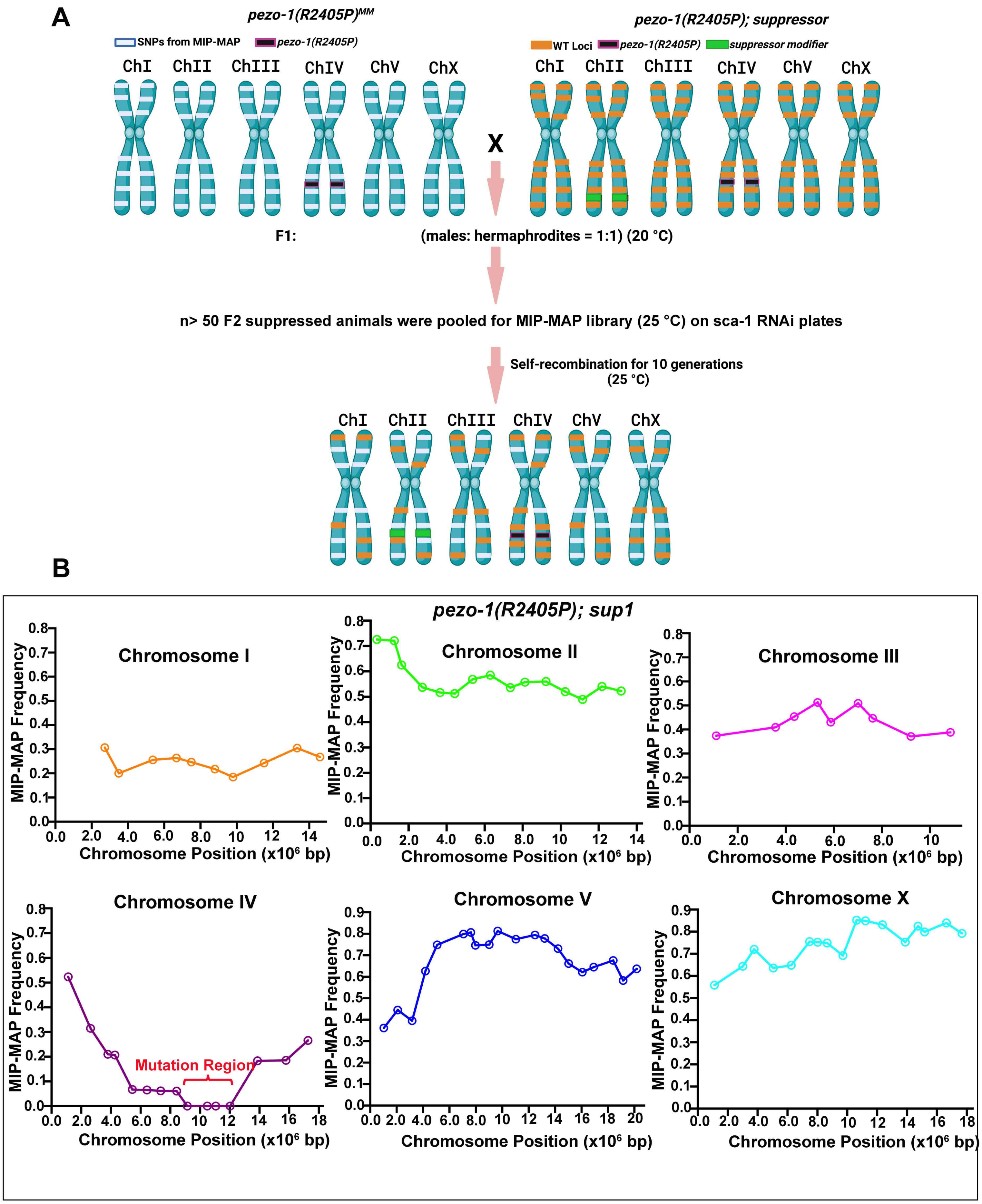

Fig. S2. Mapping of *pezo-1(R2405P)* suppressor via VC20019 and MIP-MAP sequencing.
(A) Diagram of the MIP-MAP workflow to map the single nucleotide variants (SNVs) in the suppressor line. (B-F) The read frequency of the VC20019-specific SNVs across the genome of the pooled F2 progeny from *pezo-1(R2405P); sup1* and *pezo-1(R2405P)*^{MM} cross. (B) A mutation-associated interval was identified on Chromosome IV. The strategy was generated with BioRender. com.

Development • Supplementary information

Supplemental Figure 3

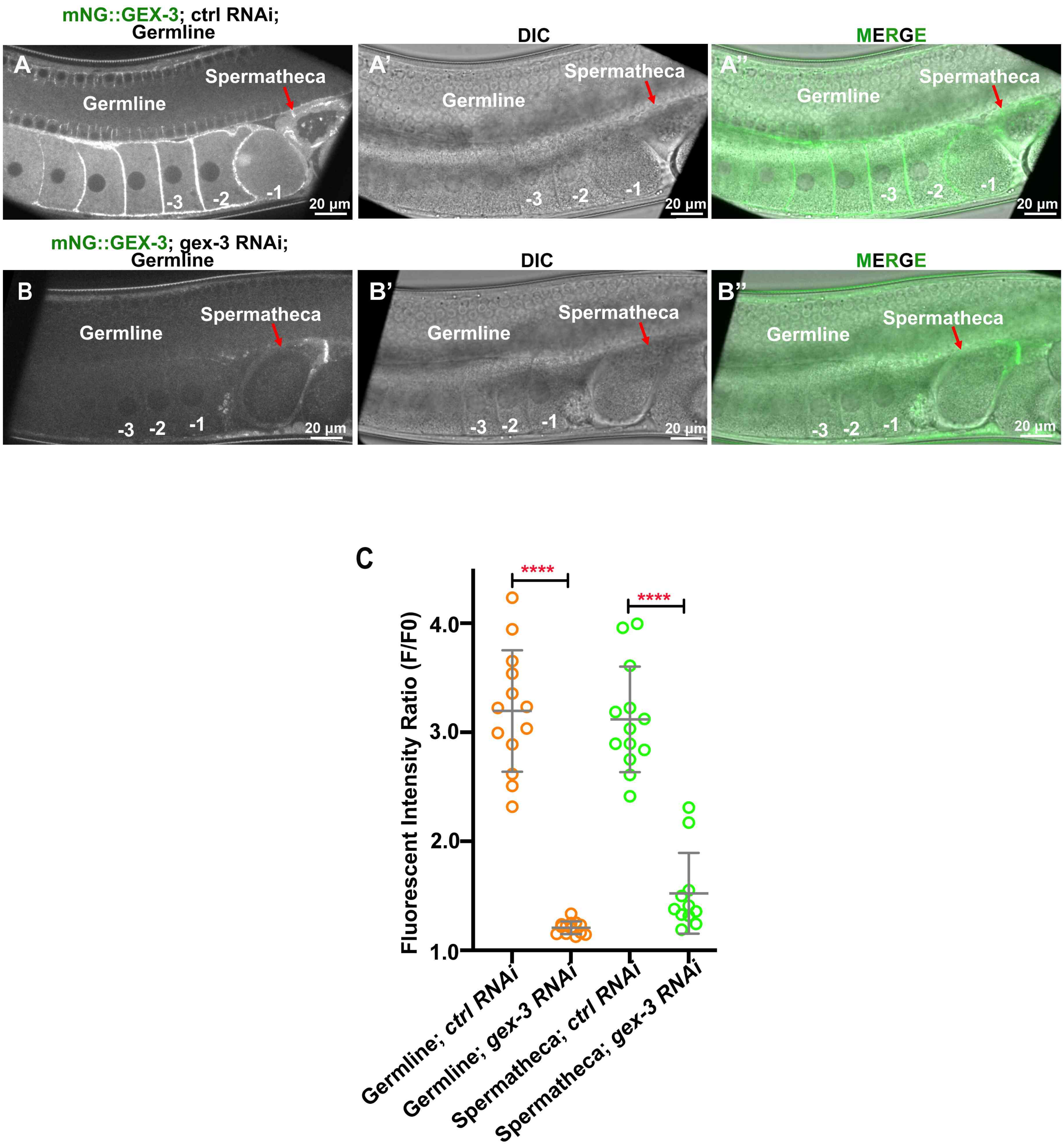

Fig. S3. ***gex-3*** **RNAi is specific and efficient to reduce the expression of** ***gex-3*** **in the germline and somatic tissue**

(A-A'') mNG::GEX-3 (green in A'') was strongly expressed on germline and oocyte membranes, and in the spermatheca (red arrowheads in A-A''). (B-B'') Depletion of *gex-3* by RNAi significantly reduced the expression of mNG::GEX-3 on the germline and oocyte membranes and in the spermatheca. DIC images are shown in panels A' and B'. (C) Quantification of fluorescent intensity of both germline and spermathecal mNG::GEX-3 with ctrl and *gex-3* RNAi. N values indicated the number of the quantified animals in (C). Scale bars are indicated in each panel. P-values: ****, p<0.0001 (t-test).

Development • Supplementary information

Supplemental Figure 4

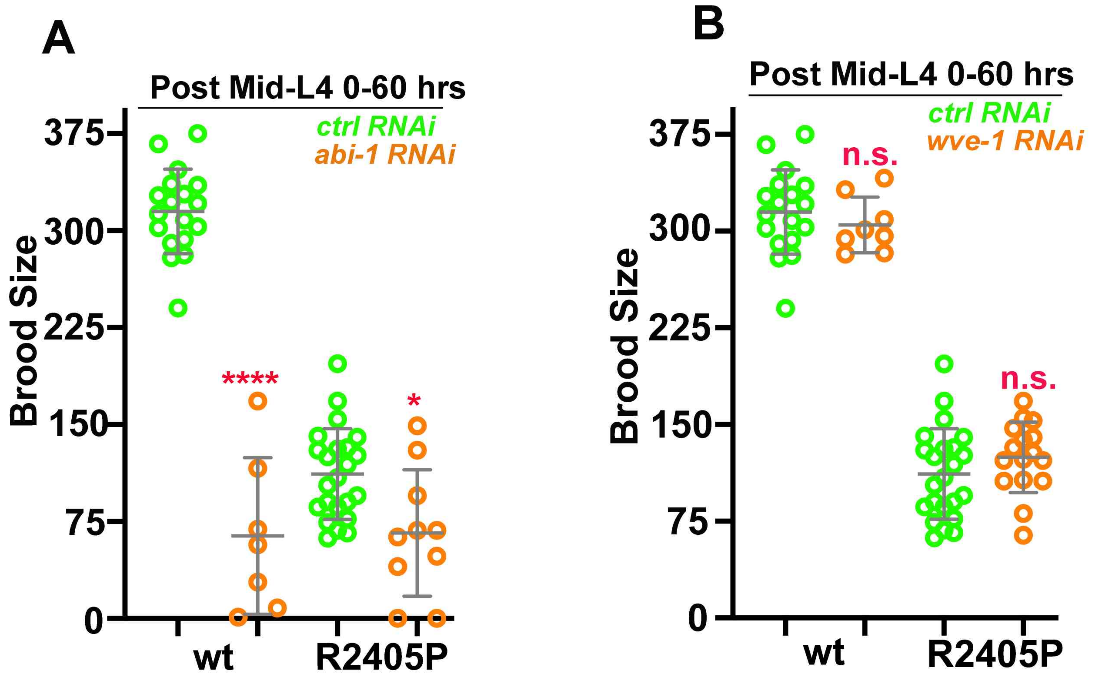

Fig. S4. Other WAVE complex subunits did not suppress the small brood size in *pezo-1(R2405P)* mutants
(A-B) 0-60 hours brood size of wild type and *pezo-1(R2405P)* animals after feeding control, abi-1 (A), and *wve-1* (B) RNAi. N values indicated the number of the tested animals in (A-B)

Development • Supplementary information

Supplemental Figure 5

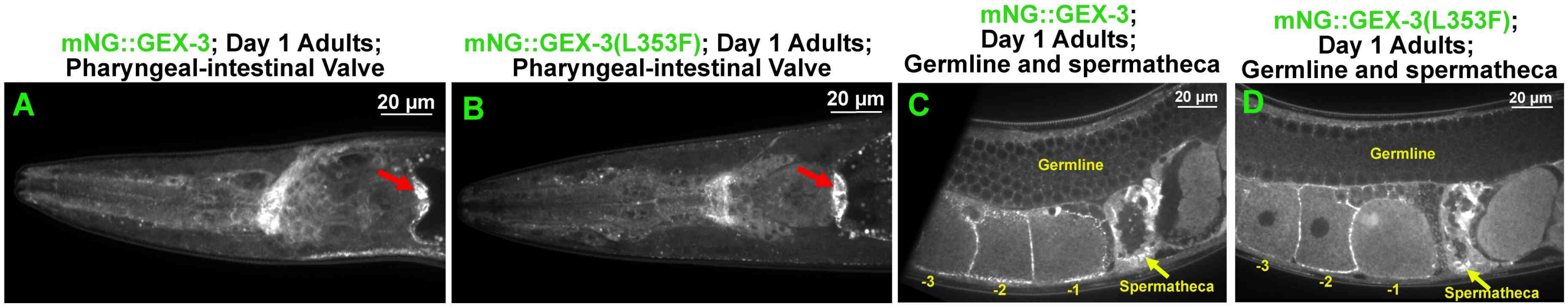

Fig. S5. mNG::GEX-3(L353F) did not disrupt the cellular localization of mNG::GEX-3

mNG::GEX-3(L353F) (B and D) presents an identical expression pattern as mNG::GEX-3(+) in a variety of cell types, including the pharyngeal-intestinal valve (red arrows in A-B), germline cells and spermathecal cells (yellow arrows in C-D). Scale bars are indicated in each panel.

Development • Supplementary information

Supplemental Figure 6

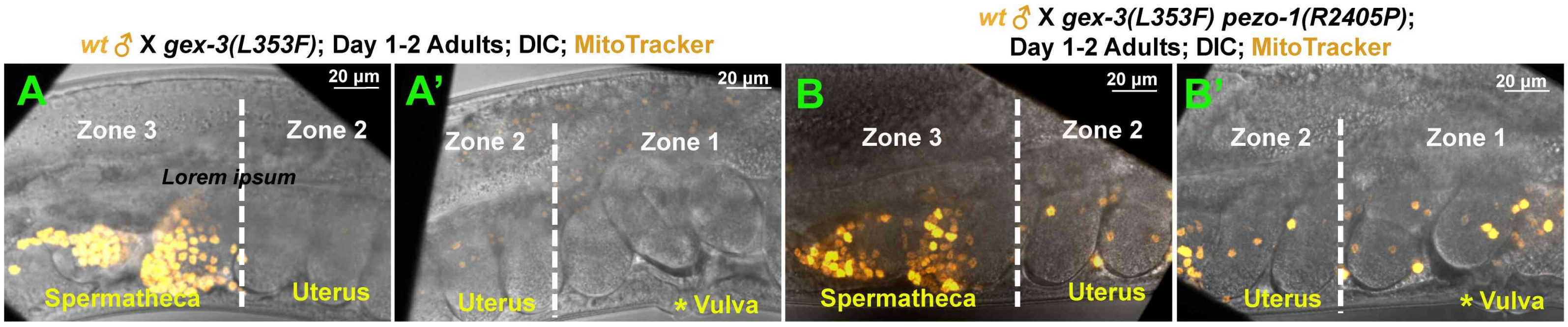

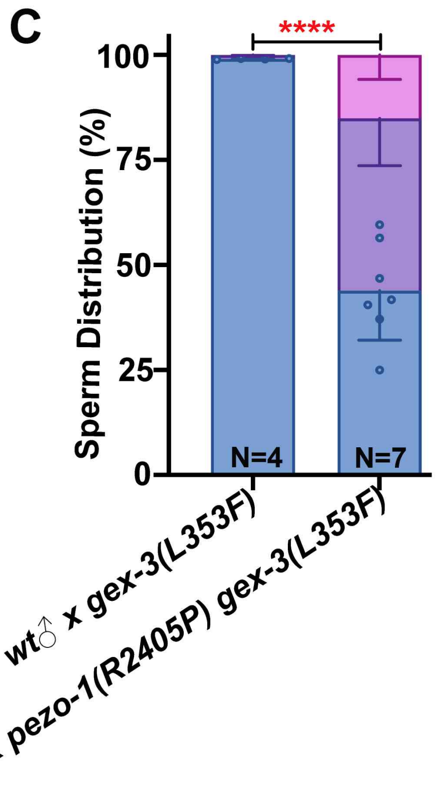

Fig. S6. *gex-3(L353F)* did not rescue sperm attraction defects in the *pezo- 1(R2405P)* mutant

(A-A') Sperm distribution was not affected in the *gex-3(L353F)* mutant. (B-B') Double mutant *pezo-1(R2405P) gex-3(L353F)* showed sperm attraction defects identical to *pezo-1(R2405P)* (Fig.2 E-E'). (C) Quantification of sperm distribution values of *gex-3(L353F)* and *pezo-1(R2405P) gex-3(L353F)* double mutant. P-values: ****, p<0.0001 (t-test). Yellow asterisks indicate the vulva (A' and B'). Scale bars are indicated at top right in each panel.

Development • Supplementary information

Supplemental Figure 7

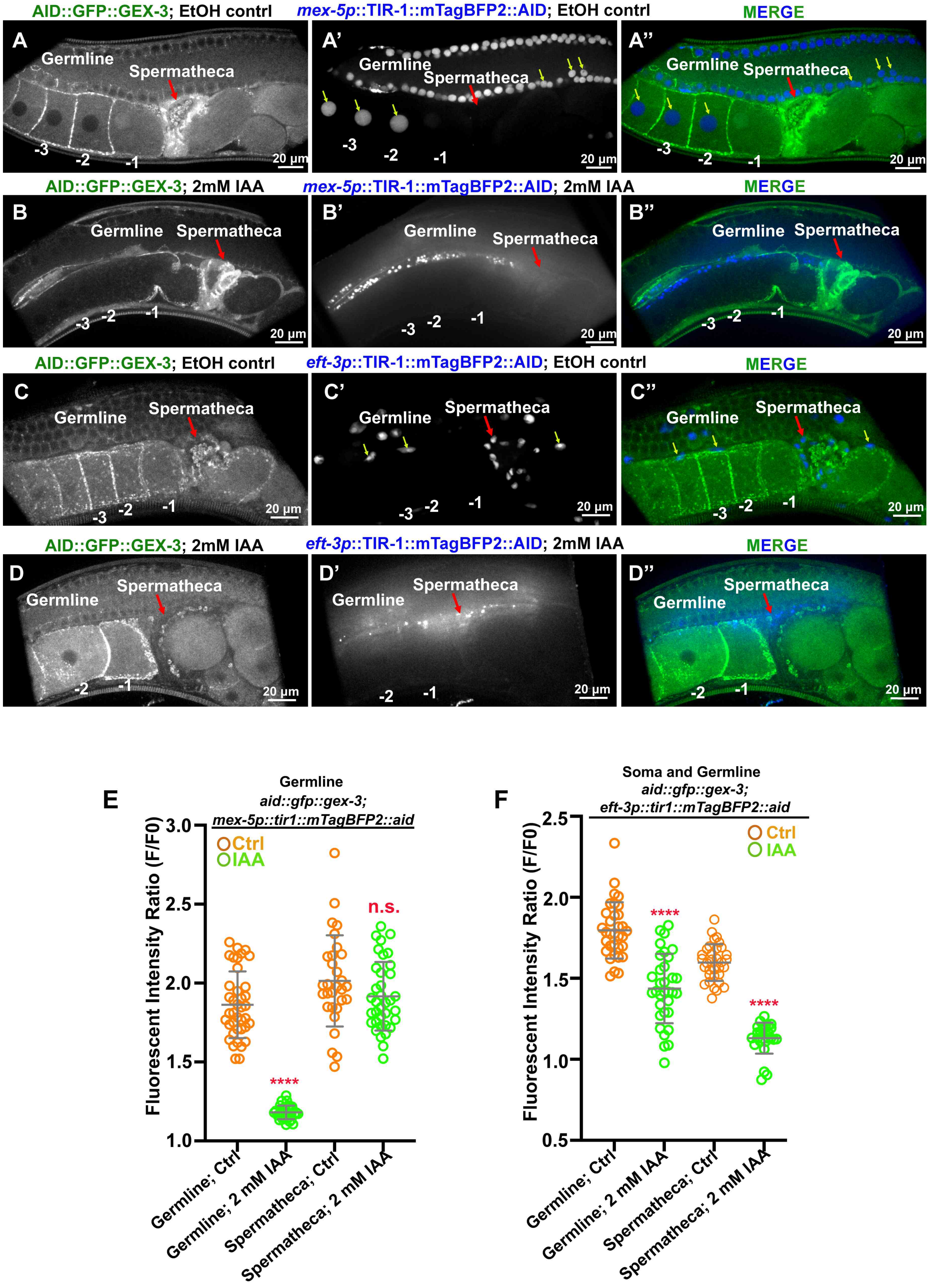

Fig. S7. Tissue-specific degradation of GEX-3 displays a reduced mNG::GEX-3 fluorescence in each tissue expressing TIR-1::mTagBFP2::AID

(A–D″) AID::mNG::GFP::GEX-3 localized to reproductive tissues, such as the plasma membrane of the germline cells, oocytes, and spermatheca (red arrows, A, A″, C, C’’). (A–A″) TIR-1::mTagBFP2::AID driven by the germline-tissue-specific promoter mex-5 is strongly expressed in the germline and oocyte nuclei (B, B’’) Fluorescent signals of AID::mNG::GEX-3 at the germline and oocyte membrane were significantly reduced when animals were treated with 2 mM auxin. However, the fluorescent signals of AID::mNG::GEX-3 at somatic spermatheca and sheath cells are not affected (B-B’’). The signal of TIR-1::mTagBFP2::AID was diminished when exposed to the auxin, regardless of the promoters were used to drive its expression (A’, B’, C’, and D’). (C-C’’) TIR-1::mTagBFP2::AID driven by the somatic specific promoters *eft-3* is strongly expressed in the nuclei of all or most somatic tissues. (D-D’’) Fluorescent signals of AID::mNG::GEX-3 driven by *eft-3* promoter at spermatheca and somatic sheath cells are significantly reduced when animals were treated with 2 mM auxin. However, fluorescent signals of AID::mNG::GEX-3 driven at germline cells, oocyte and sperm are not affected. (E–F) Quantification of the fluorescent signals of AID::mNG::GEX-3 under the different conditions. N values indicated the number of the quantified regions in (E-F). P-values: ****, $p<0.0001$ (t-test).

Table S1. *C. elegans* strains list in the study

	Strain	Genotype
Figs 1-2, and 4	AG433	*pezo-1(av162[T1997M]) IV* CRISPR/Cas9 edit
	AG434	*pezo-1(av163[R2373H]) IV* CRISPR/Cas9 edit
	AG436	*pezo-1(av162[R2405L]) IV* CRISPR/Cas9 edit
	N2	Bristol (wild-type)
	AG437	*pezo-1(av165[R2405P]) IV* CRISPR/Cas9 edit
	AG442	*pezo-1(av168[R2405Q]) IV* CRISPR/Cas9 edit
Fig. 5	LP363	*gex-3(cp114[mNG-C1^3Xflag::gex-3]) IV* CRISPR/Cas9 edit
	AG404	*pezo-1(av142 [mScarlet::pezo-1]) IV* CRISPR/Cas9 edit
Fig. 6	AG658	*gex-3(av259[L353F]) IV* CRISPR/Cas9 edit
	AG662	*pezo-1(av165[R2405P]) IV gex-3(av259[L353F]) IV*
	AG437	*pezo-1(av165[R2405P]) IV* CRISPR/Cas9 edit
	N2	Bristol (wild-type)
Fig. 7	AG699	*gex-3(av274[AID::gex-3]) IV* CRISPR/Cas9 edit
	AG713	*gex-3(av282[AID::GFP::gex-3]) IV* CRISPR/Cas9 edit
	AG714	*gex-3(av282[AID::GFP::gex-3]) IV; wrdSi23 [eft-3p::TIR1::F2A::mTagBFP2::AID*::NLS::tbb-2 3'UTR] (I:-5.32)*
	JDW225	*wrdSi23 [eft-3p::TIR1::F2A::mTagBFP2::AID*::NLS::tbb-2 3'UTR] (I:-5.32)*
	AG715	*gex-3(av282[AID::GFP::gex-3]) IV; wrdSi51 [mex-5p::TIR1::F2A::mTagBFP2::AID*::NLS::tbb-2 3'UTR] (II:0.77)*
	JDW223	*wrdSi51 [mex-5p::TIR1::F2A::mTagBFP2::AID*::NLS::tbb-2 3'UTR] (II:0.77)*
	N2	Bristol (wild-type)
Fig. 8	UN1502	*xbIs1502 [fln-1p::gfp::act-1; rol-6]*
	AG706	*gex-3(av259[L353F]) IV; xbIs1502 [fln-1p::gfp::act-1; rol-6]*
	AG708	*pezo-1(av165[R2405P]) IV gex-3(av259[L353F]) IV; xbIs1502 [fln-1p::gfp::act-1; rol-6]*
	AG739	*pezo-1(av240) IV; xbIs1502 [fln-1p::gfp::act-1; rol-6]*
Fig. S1	N2	Bristol (wild-type)
	AG437	*pezo-1(av165[R2405P]) IV* CRISPR/Cas9 edit
	AG667	*pezo-1(av165[R2405P]) IV* CRISPR/Cas9 edit; sup-1
Fig. S2	AG667	*pezo-1(av165[R2405P]) IV* CRISPR/Cas9 edit; sup-1

Development • Supplementary information

	Strain	Genotype
	VC20019	Million mutation project strain
	AG665	*pezo-1(av261[R2405^{MM}].* CRISPR/Cas9 Edit in VC20019
Fig. S3	LP363	*gex-3(cp114[mNG-C1^3Xflag::gex-3]) IV* CRISPR/Cas9 edit
Fig. S4	N2	Bristol (wild-type)
	AG437	*pezo-1(av165[R2405P]) IV* CRISPR/Cas9 edit
Fig. S5	LP363	*gex-3(cp114[mNG-C1^3Xflag::gex-3]) IV* CRISPR/Cas9 edit
	AG660	*gex-3(av260[mNG::GEX-3(L353F)] IV* CRISPR/Cas9 edit
Fig. S6	N2	Bristol (wild-type)
	AG658	*gex-3(av259[L353F]) IV* CRISPR/Cas9 edit
	AG662	*pezo-1(av165[R2405P]) IV gex-3(av259[L353F]) IV*
Fig. S7	AG714	*gex-3(av282[AID::GFP::gex-3]) IV; wrdSi23 [eft-3p::TIR1::F2A::mTagBFP2::AID*::NLS::tbb-2 3'UTR] (I:-5.32)*
	AG715	*gex-3(av282[AID::GFP::gex-3]) IV; wrdSi51 [mex-5p::TIR1::F2A::mTagBFP2::AID*::NLS::tbb-2 3'UTR] (II:0.77)*

Table S2. List of the sequence for the CRISPR design

Strain	Genotype	Description	Sequence Name	Sequence 5'-3'
AG433	*pezo-1(av162[T1997M]) IV*	Generate a point mutation T1997M in *pezo-1*	crRNA	GTACAGCTATTGATTGGACA
			Repair Template	agATTCATGAATATTCCATTCCTCTTCGAGCTTCGAACTGCAATCGACTGGACTTGGATGGATACTTCAATGCCTCTTTTCGATTTCTTCAATAT
AG434	*pezo-1(av163[R2373H]) IV*	Generate a point mutation R2373H in *pezo-1*	crRNA	AAGATTCCACGAACCAGACC
			Repair Template	TCTGTGAACATGACAGTGCTTGGAGATGTGGTGAAAATACCATGGACAAGACctggaagaaaatgagactggtaagaatttcctaa
AG436	*pezo-1(av162[R2405L]) IV*	Generate a point mutation R2405L in *pezo-1*	crRNA	CTATTTGGTTCGAGAAGCGA
			Repair Template	GATCATCTTCTCAAAATTTGTCTCGACATCTATTTAGTACTAGAAGCGAAAGACTTCATGTTGGAGCAGgtaattatttagtttta
AG442	*pezo-1(av168[R2405Q]) IV*	Generate a point mutation R2405Q in *pezo-1*	crRNA	CTATTTGGTTCGAGAAGCGA
			Repair Template	CATCTTCTCAAAATTTGTCTCGACATCTATTTGGTGCAGGAAGCGAAAGACTTCATGTTGGAGCAGgtaattatttagtttta
AG658	*gex-3(av259[L353F]) IV*	Generate a point mutation L353F in *gex-3*	crRNA	CTTTTACTACGAGATCAGCC
			Repair Template	GCCGCTTCCTTAGAAGTTCTCTTCGAGAGCTGTCATTTCTCCTCCGTGACCAACCCGGACTACTTGGC CCGAAAATTCTCTATGTCTG
AG699	*gex-3(av274[AID::gex-3]) IV*	Knock in Degron sequence at N-terminus of *gex-3*. Degron was amplified from plasmid pK0132	crRNA	TAAGCCATtttgctgaaaat
			Repair Template F1	ttgtgactcatttccccttcaattttctctccaattttcagcaaaATGGACTACAAAGACCATGACGG
			Repair Template R1	TCAGCAATTTTCACCTGTCTGGCATCTTTGTAAGCcgatgctcctgaggctcccgatgctccCTTCACGAACGCCGCCGCCT
AG713	*gex-3(av282[AID::GFP::g*	Knock in GFP sequence at N-	crRNA	GCGTTCGTGAAGggagcatc
			Repair Template F1	GTGGCCCGGAGGCGGCGGCGTTCGTGAAGagtaaaggagaagaattgtt

Development • Supplementary information

Strain	Genotype	Description	Sequence Name	Sequence 5’-3’
	ex-3]) IV	terminus of *AID::gex-3*. GFP was amplified from plasmid pDD282	Repair Template R1	GCATCTTTGTAAGCcgatgctcctgaggctcccgatgctccCTTGTAGAGCTCGTCCATT

Uppercase letters represent the ORF or exon sequence; lowercase letters indicate the sequence from intron.
