# Supplementary material for: A mutation in F-actin polymerization factor suppresses the distal arthrogryposis type 5 PIEZO2 pathogenic variant in Caenorhabditis elegans
Source: Development. 2024 Feb 13;151(4):dev202214. doi: 10.1242/dev.202214 (PMC10911111; doi:10.1242/dev.202214)
Supplement: Supplementary file 1 [file develop-151-202214_review_history.pdf]

Mutation in F-actin polymerization factor suppresses the distal arthrogryposis type 5 PIEZO2 pathogenic variant in *Caenorhabditis elegans*

Xiaofei Bai, Harold E. Smith, Luis O. Romero, Briar Bell, Valeria Vasquez and Andy Golden
DOI: 10.1242/dev.202214

Editor: Swathi Arur

Review timeline
Original submission: 24 July 2023
Editorial decision: 18 August 2023
First revision received: 21 December 2023
Accepted: 15 January 2024

Original submission

First decision letter

MS ID#: DEVELOP/2023/202214

MS TITLE: Mutation in F-actin Polymerization Factor Suppresses Distal Arthrogryposis Type 5 (DA5) PIEZO2 Pathogenic Variant in *Caenorhabditis elegans*

AUTHORS: Xiaofei Bai, Harold E. Smith, Luis O Romero, Briar Bell, Valeria Vasquez, and Andy Golden

I have now received all the referees' reports on the above manuscript, and have reached a decision. The referees' comments are appended below, or you can access them online: please go to BenchPress and click on the 'Manuscripts with Decisions' queue in the Author Area.

As you will see, the referees express considerable interest in your work, and also provide recommendations to improve the clarity and the rigor of the manuscript. In particular, recommendations of reviewer 1 and 2 pertaining to imaging PEZO-1(gf) location and levels with and without gex-1(RNAi) and in the suppressor mutant will be highly useful. In addition, all three reviewers provide detailed comments to improve the model presented and provide recommendations for textual clarifications which will add to the rigor of the study. If you are able to revise the manuscript along the lines suggested, which may involve further experiments, I will be happy receive a revised version of the manuscript.

Please attend to all of the reviewers' comments and ensure that you clearly highlight all changes made in the revised manuscript. Please avoid using 'Tracked changes' in Word files as these are lost in PDF conversion. I should be grateful if you would also provide a point-by-point response detailing how you have dealt with the points raised by the reviewers in the 'Response to Reviewers' box. If you do not agree with any of their criticisms or suggestions please explain clearly why this is so.

Reviewer 1

Advance summary and potential significance to field

© 2024. Published by The Company of Biologists under the terms of the Creative Commons Attribution License (https://creativecommons.org/licenses/by/4.0/).

This paper characterizes several disease-relevant alleles of PIEZO in the C. elegans mutant and describes the isolation of the cytoskeletal regulator GEX-3/WAVE as a suppressor of the fertility defects in the pezo-1(gf) mutant. The interaction of PIEZO with cytoskeletal regulators should be of broad interest in the mechanotransduction community, and the paper may help to explain defects seen in PIEZO-related human disorders.

Comments for the author

Line 43: The results do not show that the cytoskeleton regulates PIEZO channel activity per se (ie. Ion flux). I recommend restating this claim.

Although both PEZO-1 and GEX-1 do have mild effects on the actin cytoskeleton the paper does not directly address HOW loss of gex-3 rescues PEZO-1(gf) mutants.
Defects that lead to lower expression and/or membrane localization of PEZO-1 are possibilities for the suppression of this hyperactive allele. The authors should image PEZO-1(gf) location and levels with and without gex-1(RNAi) and in the suppressor mutant.

I recommend applying statistical correction when multiple comparisons are made.

Fig. 5, are GEX-3 and PEZO-1 colocalized? They are both at the membrane but there appears to be a 'beads on a string' appearance with alternating GEX-3 and PEZO-1 (especially in 5C). GEX-3 also seems to be expressed generally throughout the cells. Perhaps quantification of colocalization would help?

Fig. 8 'Missing Actin' where entire cells are dark (8C) isn't a phenotype specific to gex. That line sometimes silences (shows mosaic silencing) under a wide variety of different conditions. Strongly recommend removing Fig. 8J, and mention of missing actin from Fig. 9.

Editorial and minor suggestions:
General: Delete 'have shown that' and 'have been found to' etc., to reduce wordiness.
Use spermathecal when it's an adjective (e.g. spermathecal contraction).

Line 37: Restate in the positive 'significantly increased brood size and ovulation rate'
Line 39: Improve clarity, succinctness. Perhaps 'Expression of GEX-3 in the soma is required to rescue the brood size defects in pezo-1(R2405P) animals'.
Line 40: Delete 'Additionally'
Line 74: contractility (prefer to 'contractability')
Line 75: channel (not channels); Line 76 delete 'the'
Line 84: delete 'including'
Line 97: ortholog 'of' human PIEZO; delete '(pezo-1 R2405P)'
Line 116: delete 'the' before ovulation This section: Clarify how you know they are oocytes when they are crushed. In other words, are they unfertilized (no eggshell)?
Line 154: pezo-1, italicize; the 18 is a reference that doesn't seem to have been correctly added
Line 166: recommend delete 'as PEZO-1 takes longer to stop conducting cations inside the cells'
Line 188: delete 'were able to' and just say 'identified'; delete 'the' before genetic modifiers Line 210: fluorescence intensity Line 211: Delete 'The fluorescence signals of' and just say mNG::GEX-3 levels Line 213: Replace 'sufficient and specific' with 'effective'
Line 215: Delete ' to validate its suppression'.
Line 225: in (not on) the pezo-1 background Line 248: is (not was)
Line 256: list the spermatheca too Line 261: I think 'rescued' will be more clear than 'restored'
Lines 275 – 283: is nicely stated but a bit repetitive with the previous material in the paragraph
Line 439: Progeny 'were'
Line 469-470: 'unless otherwise indicated'
Line 474: Unclear what 'listed below' refers to – cite Table 3 instead.
Line 903: Partial depletion Line 910: 'By grey color' could be changed to 'in grey'

Fig. 1 (and other figures, generally): Some of the font sizes are pretty small.
To save space, it may not be necessary to list % in the graph, maybe, and you could list N below the genotypes. I prefer a sans serif font for readability. The level of statistical significance can be listed in the figure legend.

© 2024. Published by The Company of Biologists under the terms of the Creative Commons Attribution License (https://creativecommons.org/licenses/by/4.0/).

Fig. 8 typo Parallel (not Paralle)

Reviewer 2

Advance summary and potential significance to field

Summary and recommendation The authors used genome editing to create mutations in the C. elegans PIEZO mechanosensitive channel that mimic mutations seen in the human homologue associated with disease. These mutations decreased progeny production in worms likely due to defects in ovulation, “oocyte crushing”, and sperm migratory behavior likely associated with spermatheca function and PIEZO activity. The authors then carried out a suppressor screen to find interactions that could be informative to understanding PIEZO function and associated disease mechanisms. The authors identify gex-3 as a suppressor that is a component of the WAVE/SCAR complex that regulates actin dynamics. This is an exciting discovery as it links the cytoskeleton to the regulation of PIEZO channel activity. This is a very comprehensive and impressive study that provides new insights to this class of mechanosensitive channel. I am happy to recommend publication of this work in Development. My specific comments and questions are below. My primary question concerns clarity with regards to the suppressor screen mutation(s) and the relationship to gex-3 (see Experimental Approach section of this review).

Comments for the author

Experimental approach
Since mutations in human PIEZO channels are associated with disease the authors goal is to gain mechanistic insight by studying the C. elegans homologue (pezo-1) and its interactions. Four human disease sequence variants were introduced into worm pezo-1 with CRISPR/Cas9 mediated genome editing and all “human disease” or “pathogenic” variants resulted in reduced brood size, and altered reproductive tissue function resulting in ovulation defects, oocyte crushing, and disrupted sperm guidance. A specific pathogenic allele pezo-1(R2405P) is likely a gain of function mutation based on electrophysiological analysis. PEZO-1(R2405P) appears to open more easily and inactivate more slowly. To many geneticists, gain of function mutations are great starting points of suppressor screens and this R2405P mutation was used for an EMS mutagen-based suppressor screen. The pezo-1(R2405P) suppressor was mapped to a region of chromosome IV of the worm genome. A reasonable assumption for a gain of function suppressor screen is that suppressors will be loss of function and can be mimicked by RNAi. RNAi knockdown. RNAi of gex-3 in the pezo-1(R2405P) background increased brood size and depleted mNG::GEX-3 levels. Other WAVE/SCAR components did not suppress pezo-1(R2405P) reduced broods. mNG::GEX-3 also colocalized with mScarlet::PEZO-1 including the spermatheca. A CRISPR/Cas9 gex-3 mutation (L353F) also suppresses pezo-1(R2405P) phenotypes while apparently not altering GEX-3 localization patterns and levels. gex-3 RNAi knockdown in somatic tissue (spermatheca or gonad sheath cells) suppresses pezo-1(R2405P) suggesting this is an important tissue focus of this interaction and these tissues are highly contractile and dynamic with regards to ovulation and egg movement in the worm reproductive tract. Finally, the authors find that actin distribution is altered in pezo-1 mutants. This leads the authors to propose a model unifying actin regulation to the PEZO function and reproductive tract impacts on fertility.

Major considerations
Was gex-3 sequenced in the suppressor strain? Is it L353F? I follow the jump from suppressor mapping to RNAi of candidates in the region, to gex-3 RNAi suppression of pezo-1(R2405P). Is it know what the EMS induced suppressor mutation is? It would be nice to state this clearly if it is not. Discussion line 373: “Our chemical mutagen-mediated forward genetic screening …..facilitated the discovery of the suppressor alleles.” One is RNAi knockdown and one is the gex-3(L353F) allele. What is the actual suppressor from the screen?

Minor considerations, wording, and typos
Line 192 and elsewhere MM line 539, no need for extra adjectives “We pooled suppressed F2 progeny…”. Delete “carefully”.

© 2024. Published by The Company of Biologists under the terms of the Creative Commons Attribution License (https://creativecommons.org/licenses/by/4.0/).

Line 214, It would be better to say "…gex-3 RNAi was sufficient for depletion of gex-3 gene expression in vivo". You could also use the term "knockdown". RNAi is specific based on sequence homology and "deletion" is not applicable to RNAi experiments. RNAi phenocopies sequence altering mutations and does not delete any sequence.

Reviewer 3

Advance summary and potential significance to field

Andy Golden's lab pioneered the use of C. elegans to study human genetic diseases. In this study by Dr. Golden and colleagues, they turn their attention to the complex PIEZO channel. PIEZO mutation, R2405P, in humans causes distal arthrogryposis type 5. The same mutation engineered, using CRISPR, into C. elegans pezo-1, was also found to be gain of function. After studying the effects on brood size, ovulation and embryonic viability, the authors were able to conduct a suppressor screen taking advantage of the strong decrease in brood size. One suppressor turned out to be a subtle point mutation in the WAVE complex component gex-3. Auxin-inducible degradation of GEX-3 showed GEX-3 depletion from the soma could restore brood size in the pezo-1[R2405P] mutants.

Since gex-3 is known to regulate branched actin, they analyzed the effects on F-actin of the pezo-1 mutations. Intriguingly, the pezo-1 mutants have disrupted organization and orientation of actin fibers in the spermatheca, the specialized contractile tubular structure that regulates fertilization. Loss of gex-3 could partially restore the actin organization. The findings strongly support a previously unexplored role for branched F-actin in the regulation of PIEZO channel activity.

The strengths of this study include the open-ended genetic approach that identified branched actin to PIEZO channel regulation. In addition, this study uses cutting edge techniques, like CRISPR, and the auxin-inducible system applied to C. elegans. to fully explore the connection between precise mutations in PIEZO and partial loss of one branched actin regulator. Electrophysiological analysis of PEZO-1 mutants in Sf9 cell lines allows them to compare the effects of the human mutations on the C. elegans PEZO-1 channel. High throughput genetic mapping strategies allowed them to map the suppressor mutation to a small region of Chromosome IV. Then they used CRISPR to engineer the pezo-1 and gex-3 mutations into various transgenic strains, and allowed them to confirm that the suppressor mutation was responsible for rescuing the specific PEZO-1 mutation.

Comments for the author

Writing Suggestions:

p. 8 line 198, missing word "bearing the putative." putative mutation?

Figures

Overall: Some of the fonts used in Figures are too tiny.
Also, not sure what Development prefers, but these do not appear to be sans serif fonts.
For example, Fig. 3, great data but D-F, lots of white space so why is the font so small?
Fig. 7: some tiny fonts are tough to read.

Figure 8, small color-coded inserts: show Z depth, but what is this thought to show, does it matter what orientation is shown?
Maybe if the authors explain better what is happening in WT, we can better interpret the mutant changes.

Figure 9:
Is there evidence that PIEZO links to linear F-actin (as shown), as opposed to branched (which makes more sense, given the findings)?

"Perpendicular actin" is confusing. Could this be a "polarity" problem? What molecules could be providing cues to anchor actin fibers circumferentially, as opposed to "perpendicular," which may

© 2024. Published by The Company of Biologists under the terms of the Creative Commons Attribution License (https://creativecommons.org/licenses/by/4.0/).

be “random” orientation. For example, did the authors look at Cadherin? Does that have polarized distribution in the ST to anchor PEZO-1?

GEX-1 should be re-labeled WVE-1 (it was renamed some years ago).
Patel et al., 2008. doi: 10.1016/j.ydbio.2008.09.023.

Supplemental Fig. 2A: Try a color for the suppressor much different from the color for pezo-1(R2405P) so people can see the two colors. Green maybe?

Supplemental Fig. 5 B: Is it possible the Nerve Ring signal is down?
Not asking for anything here. Just a suggestion to check.

Supplemental Fig. 7.
Beautiful figure that shows depletion of GEX-3 at the Spermatheca when they use Peft-3 driver.

Additional comments/ suggestions for authors:

Regarding, why did RNAi of abi-1 and wve-1 not suppress?
Do the authors think GEX-3 has a unique role, OR could this simply be due to low RNAi penetrance? gex-3 RNAi is much more powerful than RNAi for abi-1 or wve-1, for reasons that are not understood.

F-actin phenotypes: Are there known roles for PIEZO to explain bundling or missing actin phenotypes?

First revision

Author response to reviewers' comments

We thank all reviewers for their constructive feedback and comments. I will address each reviewer’s comments in detail, providing a point-by-point response. The revised portions in the manuscript have been tracked with changes for easy identification.

Reviewer 1 Advance Summary and Potential Significance to Field: This paper characterizes several disease- relevant alleles of PIEZO in the C. elegans mutant and describes the isolation of the cytoskeletal regulator GEX-3/WAVE as a suppressor of the fertility defects in the pezo-1(gf) mutant. The interaction of PIEZO with cytoskeletal regulators should be of broad interest in the mechanotransduction community, and the paper may help to explain defects seen in PIEZO-related human disorders.

We thank the reviewer’s positive feedback and insightful comments on our study.

Reviewer 1 Comments for the Author:
Line 43: The results do not show that the cytoskeleton regulates PIEZO channel activity per se (ie. Ion flux). I recommend restating this claim.

We thanked the reviewer’ comment and have restated the claim as follows “The identification of *gex-3* as a suppressor of the pathogenic variant *pezo-1(R2405P)* suggests that PIEZO coordinates with the cytoskeleton regulator to maintain F-actin network.”

Although both PEZO-1 and GEX-1 do have mild effects on the actin cytoskeleton, the paper does not directly address HOW loss of gex-3 rescues PEZO-1(gf) mutants. Defects that lead to lower expression and/or membrane localization of PEZO-1 are possibilities for the suppression of this hyperactive allele. The authors should image PEZO-1(gf) location and levels with and without gex-1(RNAi) and in the suppressor mutant.

© 2024. Published by The Company of Biologists under the terms of the Creative Commons Attribution License (https://creativecommons.org/licenses/by/4.0/).

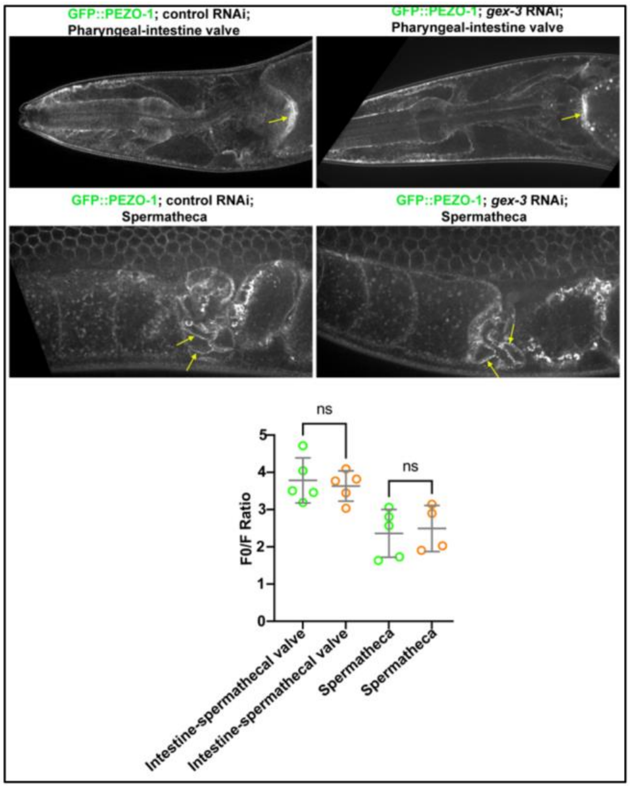

We appreciate the reviewer's constructive comment. We assessed the expression of GFP::PEZO-1 with or without *gex-3(RNAi)* (left Figure). We reveal no observable expression pattern changes or level changes in the endogenously tagged GFP::PEZO- 1 upon *gex-3* knockdown (Figure on the left). Our preliminary data (unpublished data) also indicated that the N-terminus of PEZO-1 protein, which contains the transmembrane motifs, is essential for the subcellular location and expression level of PEZO-1. While we attempted to delete the entire C- terminus motif, including the entire ion channel, did not alter the expression pattern of GFP::PEZO- 1 in somatic tissues. Therefore, we predicted that the PEZO-1(gf) mutations on the C-terminus may not impact the expressional level of endogenous PEZO-1 protein.

Combined with our physiological study and findings from human studies, the *PEZO- 1(R2405)* gain-of-function mutation decreases channel inactivation. Another study in a cancer cell line suggested that the PIEZO channel activity is essential for actin- based stress filament formation and orientation by regulating the calcium-dependent RhoA activity and other cellular signaling pathways (Pardo-Pastor, PNAS, 2018).
Therefore, we hypothesize that losing *gex-3* could lead to actin depolymerization, which provides feedback signaling to alleviate the abnormal actin mobility or orientation caused by hyperactivity of the PIEZO-1(gf) channel.
I recommend applying statistical correction when multiple comparisons are made.

We thanked the reviewer's comment and applied Tukey's tests and Bonferroni's correction after a one-way ANOVA analysis. We did not observe any significant changes in the p-value compared to the one-way ANOVA statistic test only.

Fig. 5, are GEX-3 and PEZO-1 colocalized? They are both at the membrane but there appears to be a 'beads on a string' appearance with alternating GEX-3 and PEZO-1 (especially in 5C). GEX-3 also seems to be expressed generally throughout the cells. Perhaps quantification of colocalization would help?

We appreciate the reviewer's comment. The "beads on a string pattern" was frequently observed in GEX-3 and other adherens/cadherin proteins on the plasma membrane, where these proteins form adjacent junction complexes to anchor to neighboring cells. Localization quantification of mScarlet::PEZO-1 and mNG::GEX- 3 was conducted by using the plot profile analysis (ImageJ) at

© 2024. Published by The Company of Biologists under the terms of the Creative Commons Attribution License (https://creativecommons.org/licenses/by/4.0/).

the embryonic membrane, supporting the observed colocalization pattern (see the figure below).

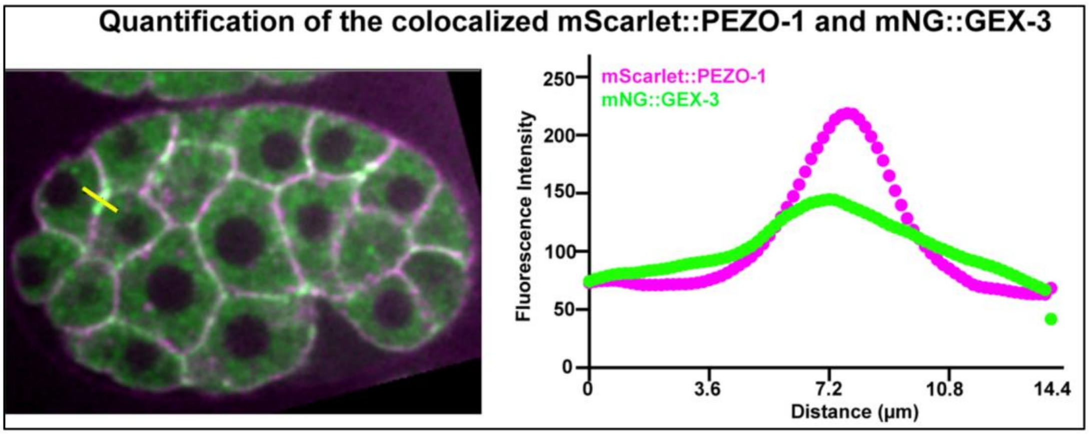

Fig. 8 'Missing Actin' where entire cells are dark (8C) isn't a phenotype specific to gex. That line sometimes silences (shows mosaic silencing) under a wide variety of different conditions. Strongly recommend removing Fig. 8J, and mention of missing actin from Fig. 9.

We thank the reviewer's comment and remove the missing actin from Figure 8.

Editorial and minor suggestions:

We thank the reviewer's comments and made changes to all the suggested comments below.
General: Delete 'have shown that' and 'have been found to' etc., to reduce wordiness. Use spermathecal when it's an adjective (e.g. spermathecal contraction).
Revised.
Line 37: Restate in the positive 'significantly increased brood size and ovulation rate'
Revised.
Line 39: Improve clarity, succinctness. Perhaps 'Expression of GEX-3 in the soma is required to rescue the brood size defects in pezo-1(R2405P) animals'.
Revised.
Line 40: Delete 'Additionally'
Revised.
Line 74: contractility (prefer to 'contractability')
Revised.
Line 75: channel (not channels); Line 76 delete 'the'
Revised.
Line 84: delete 'including'
Revised.
Line 97: ortholog 'of' human PIEZO; delete '(pezo-1 R2405P)'
Revised.
Line 116: delete 'the' before ovulation
Revised.
This section: Clarify how you know they are oocytes when they are crushed. In other words, are they unfertilized (no eggshell)?

We appreciate the reviewer's comment; nevertheless, assessing fertilization before oocytes exit the spermatheca valve presents technical challenges. The proper eggshell forms after meiotic anaphase II. Unfortunately, in the *pezo-1* mutants, oocytes/fertilized zygotes are crushed immediately after ovulation, making it difficult to evaluate eggshell integrity. Although technical constraints limit our ability to directly assess fertilization at this specific stage, our preliminary data (unpublished) on fertilization in *pezo-1* mutants revealed mild defects. Therefore, we hypothesize that many crushed oocytes may not undergo proper fertilization.

Line 154: pezo-1, italicize; the 18 is a reference that doesn't seem to have been correctly added
Revised.
Line 166: recommend delete 'as PEZO-1 takes longer to stop conducting cations inside the cells'

© 2024. Published by The Company of Biologists under the terms of the Creative Commons Attribution License (https://creativecommons.org/licenses/by/4.0/).

Revised.
Line 188: delete 'were able to' and just say 'identified'; delete 'the' before genetic modifiers
Revised.
Line 210: fluorescence intensity
Revised.
Line 211: Delete 'The fluorescence signals of' and just say mNG::GEX-3 levels
Revised.
Line 213: Replace 'sufficient and specific' with 'effective'
Revised.
Line 215: Delete ' to validate its suppression'.
Revised.
Line 225: in (not on) the pezo-1 background
Revised.
Line 248: is (not was)
Revised.
Line 256: list the spermatheca too
Revised.
Line 261: I think 'rescued' will be more clear than 'restored'
Revised.
Lines 275 – 283: is nicely stated but a bit repetitive with the previous material in the paragraph
Revised.
Line 439: Progeny 'were'
Revised.
Line 469-470: 'unless otherwise indicated'
Revised.
Line 474: Unclear what 'listed below' refers to – cite Table 3 instead.
Revised.
Line 903: Partial depletion
Revised.
Line 910: 'By grey color' could be changed to 'in grey'
Revised.
Fig. 1 (and other figures, generally): Some of the font sizes are pretty small. To save space, it may not be necessary to list % in the graph, maybe, and you could list N below the genotypes. I prefer a sans serif font for readability. The level of statistical significance can be listed in the figure legend.

Thank you for the reviewer's suggestions. We have enlarged all figures and adopted the sans-serif font Arial.

Fig. 8 typo Parallel (not Paralle)
Revised.

Reviewer 2 Advance Summary and Potential Significance to Field:
Summary and recommendation
The authors used genome editing to create mutations in the C. elegans PIEZO mechanosensitive channel that mimic mutations seen in the human homologue associated with disease. These mutations decreased progeny production in worms likely due to defects in ovulation, "oocyte crushing", and sperm migratory behavior likely associated with spermatheca function and PIEZO activity. The authors then carried out a suppressor screen to find interactions that could be informative to understanding PIEZO function and associated disease mechanisms. The authors identify gex-3 as a suppressor that is a component of the WAVE/SCAR complex that regulates actin dynamics. This is an exciting discovery as it links the cytoskeleton to the regulation of PIEZO channel activity. This is a very comprehensive and impressive study that provides new insights to this class of mechanosensitive channel. I am happy to recommend publication of this work in Development. My specific comments and questions are below. My primary question concerns clarity with regards to the suppressor screen mutation(s) and the relationship to gex-3 (see Experimental Approach section of this review).
We thank the reviewer's positive feedback and comments on our study.

Reviewer 2 Comments for the Author:

© 2024. Published by The Company of Biologists under the terms of the Creative Commons Attribution License (https://creativecommons.org/licenses/by/4.0/).

Experimental approach
Since mutations in human PIEZO channels are associated with disease the authors goal is to gain mechanistic insight by studying the C. elegans homologue (pezo-1) and its interactions. Four human disease sequence variants were introduced into worm pezo-1 with CRISPR/Cas9 mediated genome editing and all "human disease" or "pathogenic" variants resulted in reduced brood size, and altered reproductive tissue function resulting in ovulation defects, oocyte crushing, and disrupted sperm guidance. A specific pathogenic allele pezo-1(R2405P) is likely a gain of function mutation based on electrophysiological analysis. PEZO-1(R2405P) appears to open more easily and inactivate more slowly. To many geneticists, gain of function mutations are great starting points of suppressor screens and this R2405P mutation was used for an EMS mutagen-based suppressor screen. The pezo-1(R2405P) suppressor was mapped to a region of chromosome IV of the worm genome. A reasonable assumption for a gain of function suppressor screen is that suppressors will be loss of function and can be mimicked by RNAi. RNAi knockdown. RNAi of gex-3 in the pezo-1(R2405P) background increased brood size and depleted mNG::GEX-3 levels. Other WAVE/SCAR components did not suppress pezo-1(R2405P) reduced broods. mNG::GEX-3 also colocalized with mScarlet::PEZO-1 including the spermatheca. A CRISPR/Cas9 gex-3 mutation (L353F) also suppresses pezo-1(R2405P) phenotypes while apparently not altering GEX-3 localization patterns and levels. gex-3 RNAi knockdown in somatic tissue (spermatheca or gonad sheath cells) suppresses pezo-1(R2405P) suggesting this is an important tissue focus of this interaction and these tissues are highly contractile and dynamic with regards to ovulation and egg movement in the worm reproductive tract. Finally, the authors find that actin distribution is altered in pezo-1 mutants. This leads the authors to propose a model unifying actin regulation to the PEZO function and reproductive tract impacts on fertility.
We thank the reviewer's positive feedback and comments on our study.

Major considerations
Was gex-3 sequenced in the suppressor strain? Is it L353F? I follow the jump from suppressor mapping to RNAi of candidates in the region, to gex-3 RNAi suppression of pezo-1(R2405P). Is it know what the EMS induced suppressor mutation is? It would be nice to state this clearly if it is not.

We appreciate the reviewer's comments on our study. We conducted both MIP-MAP genome mapping and whole-genome sequencing to identify suppressor candidate alleles in our suppressor strain. Following MIP-MAP and whole-genome sequencing, we narrowed the candidate list to six genes, each with a missense mutation. We specifically selected homozygous mutations in the candidate strain, hypothesizing that these missense mutations exhibit a recessive loss-of-function nature. To validate each candidate, we conducted a trial RNAi screening. Our RNAi experiments confirmed that *gex-3*, one of the candidates, indeed suppressed the small brood size. To further verify the suppressive effect, we generated the *gex-3(L353F*), identified in the suppressor strain, using CRISPR/Cas9 gene editing in the context of the *pezo-1(R2405P)* mutant background. The CRISPR-edited suppressor allele is consistent with RNAi experiments in restoring a small brood in the *pezo-1(R2405P)*.

Discussion line 373: "Our chemical mutagen-mediated forward genetic screening …..facilitated the discovery of the suppressor alleles." One is RNAi knockdown and one is the gex-3(L353F) allele. What is the actual suppressor from the screen?

We appreciate the reviewer's comments. While we initially defined the suppressor as a missense mutation, namely the *gex-3(L353F)* allele, our extensive study suggests that this mutation functions as a loss-of-function allele. This conclusion aligns with our observations of successful rescue in other conditions, achieved either by knocking down *gex-3* transcriptional levels through RNAi or by degrading the GEX- 3 protein using the auxin-inducible degradation system.

Minor considerations, wording, and typos
Line 192 and elsewhere MM line 539, no need for extra adjectives "We pooled suppressed F2 progeny…". Delete "carefully".
Revised.
Line 214, It would be better to say "…gex-3 RNAi was sufficient for depletion of gex-3 gene expression in vivo". You could also use the term "knockdown". RNAi is specific based on sequence homology and "deletion" is not applicable to RNAi experiments. RNAi phenocopies sequence

© 2024. Published by The Company of Biologists under the terms of the Creative Commons Attribution License (https://creativecommons.org/licenses/by/4.0/).

altering mutations and does not delete any sequence.

We thank the reviewer's suggestions and revised the writing.

Reviewer 3 Advance Summary and Potential Significance to Field:
Andy Golden's lab pioneered the use of C. elegans to study human genetic diseases. In this study by Dr. Golden and colleagues, they turn their attention to the complex PIEZO channel. PIEZO mutation, R2405P, in humans causes distal arthrogryposis type 5. The same mutation engineered, using CRISPR, into C. elegans pezo-1, was also found to be gain of function. After studying the effects on brood size, ovulation and embryonic viability, the authors were able to conduct a suppressor screen taking advantage of the strong decrease in brood size. One suppressor turned out to be a subtle point mutation in the WAVE complex component gex-3. Auxin-inducible degradation of GEX-3 showed GEX-3 depletion from the soma could restore brood size in the pezo-1[R2405P] mutants.

Since gex-3 is known to regulate branched actin, they analyzed the effects on F-actin of the pezo-1 mutations. Intriguingly, the pezo-1 mutants have disrupted organization and orientation of actin fibers in the spermatheca, the specialized contractile tubular structure that regulates fertilization. Loss of gex-3 could partially restore the actin organization. The findings strongly support a previously unexplored role for branched F-actin in the regulation of PIEZO channel activity.

The strengths of this study include the open-ended genetic approach that identified branched actin to PIEZO channel regulation. In addition, this study uses cutting edge techniques, like CRISPR, and the auxin-inducible system applied to C. elegans. to fully explore the connection between precise mutations in PIEZO and partial loss of one branched actin regulator. Electrophysiological analysis of PEZO-1 mutants in Sf9 cell lines allows them to compare the effects of the human mutations on the C. elegans PEZO-1 channel. High throughput genetic mapping strategies allowed them to map the suppressor mutation to a small region of Chromosome IV. Then they used CRISPR to engineer the pezo-1 and gex-3 mutations into various transgenic strains, and allowed them to confirm that the suppressor mutation was responsible for rescuing the specific PEZO-1 mutation.

We thank the reviewer's positive feedback and comments on our study.

Reviewer 3 Comments for the Author:
Writing Suggestions:

p. 8 line 198, missing word "bearing the putative." putative mutation?
We thank the reviewer's careful reading and comment, and we revised the manuscript.

Figures

Overall: Some of the fonts used in Figures are too tiny. Also, not sure what Development prefers, but these do not appear to be sans serif fonts. For example, Fig. 3, great data but D-F, lots of white space so why is the font so small? Fig. 7: some tiny fonts are tough to read.

Thank the reviewer's suggestions. We enlarged all figures and used the sans serif font of Arial throughout the figures.

Figure 8, small color-coded inserts: show Z depth, but what is this thought to show, does it matter what orientation is shown? Maybe if the authors explain better what is happening in WT, we can better interpret the mutant changes.

We appreciate the reviewer's comment. The color-coded insert illustrates the spatial arrangement of F-actin filaments. More color-coded filaments indicate greater z-depth occupation. In the wild type, most filaments align parallel within 2- 3 µm, usually with one or two colors. However, in the *pezo-1* mutants, we observed a significant disturbance in this z-depth pattern, characterized by the presence of 3-5 colors (3-5 µm z-depth). This suggests that F-actin filaments not only misalign horizontally but also occupy significantly more z-depth, as indicated by the increased number of

© 2024. Published by The Company of Biologists under the terms of the Creative Commons Attribution License (https://creativecommons.org/licenses/by/4.0/).

color-coded filaments.

Figure 9:
Is there evidence that PIEZO links to linear F-actin (as shown), as opposed to branched (which makes more sense, given the findings)?

The reciprocal regulations between the PIEZO channel and the actin-based cytoskeleton are currently gaining attention in the field of mechanobiology. Recent studies indicate functional interconnections between the PIEZO channel and the actin cytoskeleton, encompassing: 1) integrin-linked actin cytoskeleton's indirect effects on plasma membrane stiffness, influencing PIEZO activity; 2) physical tethering of the PIEZO channel to the actin cytoskeleton via E-cadherin/catenin complexes, regulating PIEZO channel activity; 3) the influence of PIEZO activity on actin depolarization through Ca^{2+}-dependent RhoA and other regulators, impacting the orientation of focal adhesions and actin-based stress fibers, which are similar to linear F-actin; 4) Overexpression of cofilin (a protein that severs actin filaments) in a mouse model of Angelman syndrome decreases PIEZO2 function in sensory neurons. Therefore, significant remodeling of the actin cytoskeleton is crucial for Piezo-induced cell mobility and migration in various cell types; conversely, PIEZO activity also contributes to the dynamics and formation of the actin-based cytoskeleton (Pardo-Pastor, 2017; Nourse, 2017; Kuntze, 2020; Maneshi, 2018; Romero, 2023).

"Perpendicular actin" is confusing. Could this be a "polarity" problem? What molecules could be providing cues to anchor actin fibers circumferentially, as opposed to "perpendicular," which may be "random" orientation. For example, did the authors look at Cadherin? Does that have polarized distribution in the ST to anchor PEZO-1?

Thank you for the reviewer's insightful comments. We examined the expression pattern of DLG-1::GFP, an apical junction marker, in the spermatheca. However, no abnormal patterns were observed in the *pezo-1* null mutant compared to wt (see the figure below).

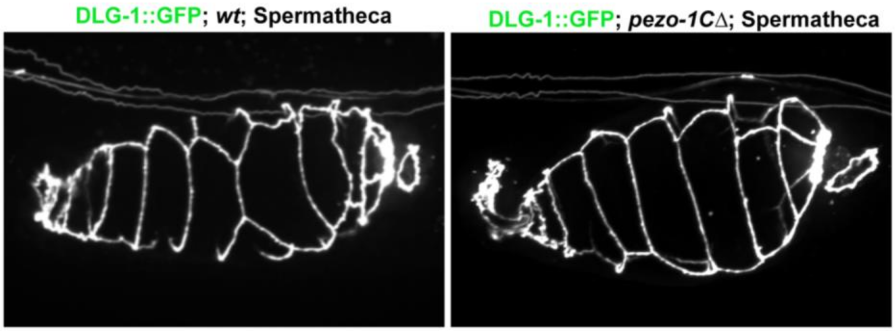

Our current hypothesis suggests that the F-actin polymerization phenotypes result from PIEZO-dependent signaling pathways that excessively depolymerize actin. The PEZO-1 gain-of-function mutant may act as a hyperactivated regulator, forming abundant shorter filaments and resulting in misaligned filaments or bundles. Knockdown of *gex-3*, aimed at reducing the formation of new filaments, could mitigate depolarization defects during actin dynamics.

While our initial prediction was that PEZO-1 might regulate signaling through calcium influx, our previous study, testing calcium influx during ovulation at the spermatheca, did not reveal dramatic calcium defects. We suspect this might be due to the insensitivity of our GCaMP3 sensor. Future studies will require highly sensitive calcium sensors and electrophysiological assays to investigate PEZO-1- dependent cellular regulators contributing to actin network formation and dynamics.

GEX-1 should be re-labeled WVE-1 (it was renamed some years ago). Patel et al., 2008. doi: 10.1016/j.ydbio.2008.09.023.

We appreciate the reviewer's updated information. We have revised the gene name to *wve-1.*

Supplemental Fig. 2A: Try a color for the suppressor much different from the color for pezo 1(R2405P) so people can see the two colors. Green maybe?

© 2024. Published by The Company of Biologists under the terms of the Creative Commons Attribution License (https://creativecommons.org/licenses/by/4.0/).

We appreciate the reviewer's comment and have incorporated the suggested changes, now reflected in the revised figure with the green color.

Supplemental Fig. 5 B: Is it possible the Nerve Ring signal is down? Not asking for anything here. Just a suggestion to check.

After reviewing the original images, it seems that the nerve ring signal appears normal compared to the wild type, likely due to the choice of the z-planes for the z-max analysis.

Supplemental Fig. 7. Beautiful figure that shows depletion of GEX-3 at the Spermatheca when they use Peft-3 driver.

We thank the reviewer's positive comments on our data.

Additional comments/ suggestions for authors:

Regarding, why did RNAi of abi-1 and wve-1 not suppress? Do the authors think GEX-3 has a unique role, OR could this be due to low RNAi penetrance? gex-3 RNAi is much more powerful than RNAi for abi-1 or wve-1, for reasons that are not understood.

We appreciate the reviewer's careful reading and comments. We meticulously titrated the RNAi conditions to knock down three genes for at least one generation before conducting the assay. We observed that *wve-1* RNAi produced the mildest phenotype by itself, whereas *abi-1* RNAi displayed the most severe phenotypes in the context of small brood size. However, we did not observe that *abi-1* RNAi suppresses the small brood size in the *pezo-1(R2405P)* mutant. We hypothesized that *gex-3* may be the specific suppressor gene for the *R2405P* mutant. However, we cannot rule out the possibility that RNAi efficiency may contribute to this conclusion. In future studies, we plan to use an auxin-degradation system to degrade these two proteins in a tissue-specific manner.

F-actin phenotypes: Are there known roles for PIEZO to explain bundling or missing actin phenotypes?

Reviewer #1 pointed out that the missing actin could be due to transgene silencing, so we removed the missing actin characterization from the figure and manuscript. Our current hypothesis is that the F-actin polymerization phenotypes result from PIEZO-dependent signaling pathways that excessively depolymerize actin. The PEZO-1 gain-of-function mutant may act as a hyperactivated regulator, forming abundant shorter filaments and resulting in misaligned filaments or bundles. Knockdown of *gex-3*, aimed at reducing the formation of new filaments, could mitigate depolarization defects during actin dynamics. Future studies will require highly sensitive calcium sensors and electrophysiological assays to investigate whether and how PEZO-1-dependent cellular regulators contribute to the actin network's orientation, formation, and dynamics.

<u>Second decision letter</u>

MS ID#: DEVELOP/2023/202214

MS TITLE: Mutation in F-actin Polymerization Factor Suppresses Distal Arthrogryposis Type 5 (DA5) PIEZO2 Pathogenic Variant in *Caenorhabditis elegans*

AUTHORS: Xiaofei Bai, Harold E. Smith, Luis O Romero, Briar Bell, Valeria Vasquez, and Andy Golden
ARTICLE TYPE: Research Article

I am happy to tell you that your manuscript has been accepted for publication in Development, pending our standard ethics checks.

© 2024. Published by The Company of Biologists under the terms of the Creative Commons Attribution License (https://creativecommons.org/licenses/by/4.0/).

Reviewer 1

Advance summary and potential significance to field

This paper identifies a key actin regulator (GEX-1, the only WAVE/SCAR protein in C. elegans) as a genetic modifier of PIEZO function.

Comments for the author

The authors have made or responded to my suggested revisions, and I am satisfied with the current version of the paper.

Reviewer 3

Advance summary and potential significance to field

Andy Golden's lab pioneered the use of C. elegans to study human genetic diseases. In this study by Dr. Golden and colleagues, they turned their attention to the complex PIEZO channel. PIEZO mutation, R2405P, in humans causes distal arthrogryposis type 5. The same mutation engineered, using CRISPR, into C. elegans pezo-1, was also found to be gain of function. After studying the effects on brood size, ovulation and embryonic viability, the authors were able to conduct a suppressor screen taking advantage of the strong decrease in brood size. One suppressor turned out to be a subtle point mutation in the WAVE complex component gex-3. Auxin-inducible degradation of GEX-3 showed GEX-3 depletion from the soma could restore brood size in the pezo-1[R2405P] mutants.

Since gex-3 is known to regulate branched actin, they analyzed the effects on F-actin of the pezo-1 mutations. Intriguingly, the pezo-1 mutants have disrupted organization and orientation of actin fibers in the spermatheca, the specialized contractile tubular structure that regulates fertilization. Loss of gex-3 could partially restore the actin organization. The findings strongly support a previously unexplored role for branched F-actin in the regulation of PIEZO channel activity.

The strengths of this study include the open-ended genetic approach that identified branched actin to PIEZO channel regulation. In addition, this study uses cutting edge techniques, like CRISPR, and the auxin-inducible system applied to C. elegans. to fully explore the connection between precise mutations in PIEZO and partial loss of one branched actin regulator. Electrophysiological analysis of PEZO-1 mutants in Sf9 cell lines allows them to compare the effects of the human mutations on the C. elegans PEZO-1 channel. High throughput genetic mapping strategies allowed them to map the suppressor mutation to a small region of Chromosome IV. Then they used CRISPR to engineer the pezo-1 and gex-3 mutations into various transgenic strains, and allowed them to confirm that the suppressor mutation was responsible for rescuing the specific PEZO-1 mutation.

The findings in this paper will lead PIEZO researchers to investigate the mechanistic interaction between branched actin and positive or negative regulation of PIEZO. Understanding how the allele-specific suppression of a gain of function PIEZO channel by loss of a branched actin regulator raises exciting questions that will require biochemical, structural, and further imaging approaches.

Comments for the author

The authors responded to my and other Reviewer questions thoroughly and adequately. I am still puzzled about what is anchoring those basal actin fibers, but it appears this has not been determined for those cells. I am sure further studies will lead to more exciting findings regarding how allele-specific rescue of the Piezo mutation was made possible by loss of branched actin.

© 2024. Published by The Company of Biologists under the terms of the Creative Commons Attribution License (https://creativecommons.org/licenses/by/4.0/).
